# Oxygen isotope fractionation during anaerobic ammonium oxidation by the marine representative *Candidatus* Scalindua sp.

**DOI:** 10.1093/ismejo/wraf115

**Published:** 2025-06-02

**Authors:** Kanae Kobayashi, Kazuya Nishina, Keitaro Fukushima, Yuji Onishi, Akiko Makabe, Mamoru Oshiki, Keisuke Koba, Satoshi Okabe

**Affiliations:** Division of Environmental Engineering, Faculty of Engineering, Hokkaido University, Sapporo, Hokkaido 060-8628, Japan; Institute for Extra-cutting-edge Science and Technology Avant-garde Research (X-star), Japan Agency for Marine-Earth Science and Technology (JAMSTEC), Yokosuka 237-0061, Japan; Biogeochemical Cycle Modeling and Analysis Section, Earth System Division, National Institute for Environmental Studies, Onogawa, Tsukuba 305-8506, Japan; Center for Ecological Research, Kyoto University, Otsu, Shiga 520-2113, Japan; Faculty of Food and Agricultural Sciences, Fukushima University, 1 Kanayagawa, Fukushima, 960-1296, Japan; Center for Ecological Research, Kyoto University, Otsu, Shiga 520-2113, Japan; Research Institute for Humanity and Nature, 457-4, Kamigamo-Motoyama, Kita-ku, Kyoto, 603-8047, Japan; Institute for Extra-cutting-edge Science and Technology Avant-garde Research (X-star), Japan Agency for Marine-Earth Science and Technology (JAMSTEC), Yokosuka 237-0061, Japan; Division of Environmental Engineering, Faculty of Engineering, Hokkaido University, Sapporo, Hokkaido 060-8628, Japan; Center for Ecological Research, Kyoto University, Otsu, Shiga 520-2113, Japan; Division of Environmental Engineering, Faculty of Engineering, Hokkaido University, Sapporo, Hokkaido 060-8628, Japan

**Keywords:** anammox, Scalindua sp, kinetic oxygen isotope effect, oxygen isotope exchange between NO_2_^−^ and H_2_O

## Abstract

Analysing the nitrogen (^15^ε) and oxygen (^18^ε) isotope effects of anaerobic ammonium oxidation (anammox) is essential for accurately assessing its potential contribution to fixed-N losses in the ocean, yet the ^18^ε of anammox remains unexplored. Here, we determined the previously unexplored ^18^ε of anammox using a highly enriched culture of the marine anammox species “*Ca.* Scalindua sp”. Because *Scalindua* significantly accelerated oxygen isotope exchange between NO_2_^−^ and H_2_O, we introduced a new rate constant for anammox-mediated oxygen isotope exchange (*k*_eq, AMX_ = 8.44 ~ 13.56 × 10^−2^ h^−1^), which is substantially faster than abiotic oxygen isotope exchange (*k*_eq, abio_ = 1.13 × 10^−2^ h^−1^), into a numerical model to estimate the ^18^ε during anammox. Based on our experimental results, we successfully determined the ^18^ε associated with: (1) conversion of NO_2_^−^ to N_2_ (*^18^ε_NO2- → N2_* = 10.6 ~ 16.1‰), (2) NO_2_^−^ oxidation to NO_3_^−^ (*^18^ε_NO2- → NO3-_* = −2.9 ~ −11.0‰, inverse fractionation), (3) incorporation of oxygen from water during NO_2_^−^ oxidation to NO_3_^−^ (*^18^ε_H2O_* = 16.4 ~ 19.2‰). Our study underscores the possibility that unique anammox oxygen isotope signals may be masked due to substantial anammox-mediated oxygen isotope exchange between NO_2_^−^ and H_2_O. Therefore, careful consideration is required when utilizing *δ*^18^O_NO3-_ and *δ*^18^O_NO2-_ as geochemical markers to assess the potential contribution of anammox to fixed-N losses in the ocean.

## Graphical Abstract

**Graphical Abstract:**
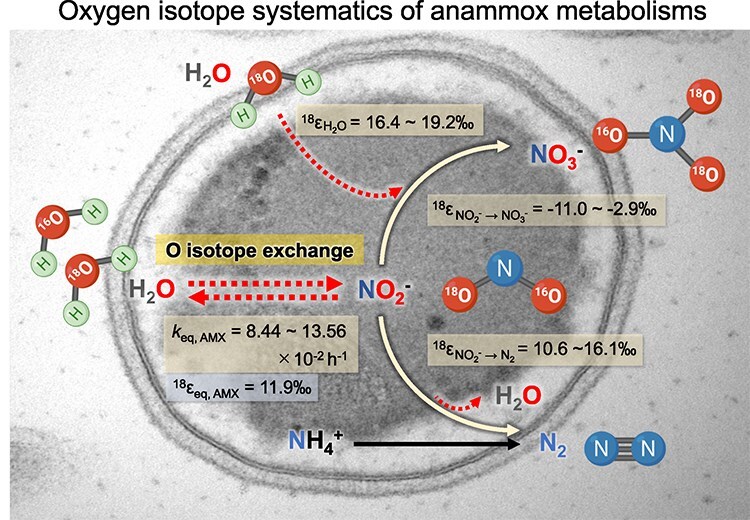

## Introduction

Anaerobic ammonium oxidation (anammox) and denitrification are the two primary sinks of fixed nitrogen in marine ecosystems. Anammox bacteria oxidize NH_4_^+^ directly to N_2_ gas using NO_2_^−^ as the terminal electron acceptor, and simultaneously oxidize NO_2_^−^ to NO_3_^−^ [1], as represented by the following stoichiometric equation [2]:


\begin{align*} &1{\mathrm{N}\mathrm{H}}_4^{+}+1.146{\mathrm{N}\mathrm{O}}_2^{-}+0.071{\mathrm{H}\mathrm{CO}}_3^{-}+0.057{\mathrm{H}}^{+}\to 0.986{\mathrm{N}}_2\\&\ \ \ +0.161{\mathrm{N}\mathrm{O}}_3^{-}+0.071{\mathrm{CH}}_{1.74}{\mathrm{O}}_{0.31}{\mathrm{N}}_{0.20}+2.002{\mathrm{H}}_2\mathrm{O} \end{align*}

The significant role of anammox bacteria in nitrogen removal has been documented in oxygen-deficient water columns [3–8] and marine sediments [9–13]. In these ecosystems, the stable isotope ratios of nitrogen (^15^N/^14^N) and oxygen (^18^O/^16^O) in reactive nitrogen compounds (e.g. NH_4_^+^, NO_2_^−,^ and NO_3_^−^) have been used as geochemical tracers to evaluate nitrogen sources and sinks [14–21] and to determine *in situ* turnover rates [22]. To quantitatively assess the impacts of microbial processes on nitrogen pools, isotope fractionation is measured using the kinetic isotope effect, defined as ε (‰) = [(*k*_L_ / *k*_H_) – 1] × 1000, where *k*_L_ / *k*_H_ represents the ratio of the first-order reaction rate constants between the light (*k*_L_) and heavy (*k*_H_) isotopically substituted substrates. The kinetic nitrogen and oxygen isotope effects (^15^ε and ^18^ε) in key microbial processes provide a fundamental basis for interpreting natural abundance nitrogen isotopic distributions in the ocean. The dual isotope effects (^15^ε and ^18^ε) associated with anammox metabolism enable a more precise assessment of its contributions to nitrogen loss.

^15^ε values for anammox metabolism have been determined for four anammox species; “*Ca.* Kuenenia stuttgartiensis” [23], “*Ca.* Scalindua japonica”, “*Ca.* Jettenia caeni”, and “*Ca.* Brocadia sinica”, all of which were cultured in continuous bioreactors [24]. Additionally, isotope effects have been measured for biomass containing anammox bacteria originating from wastewater treatment plants [25, 26].

**Figure 1:**
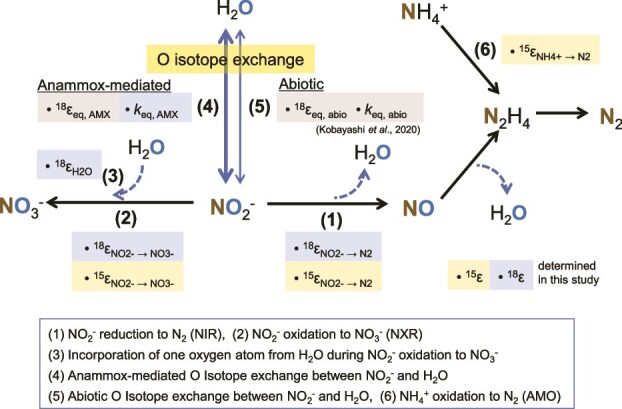
Description of anammox metabolism and associated nitrogen and oxygen isotope effects. δ^18^O_NO₂_^−^ and δ^18^_NO₃_^−^ are influenced by reaction (1), reaction (2), and the equilibrium between NO₂^−^ and H₂O, described by reactions (4) and (5). We determined the following three kinetic oxygen isotope effects (^18^ε); (1) ^18^ε_NO2- → N2_, (2) ^18^ε_NO2- → NO3-_, and (3) ^18^ε_H2O_, as well as (4) a reaction rate constant (*k*_eq, AMX_) for anammox-mediated oxygen isotope exchange between NO_2_^−^ and H_2_O. In our previous study, we determined the equilibrium isotope effect (^18^ε_eq, abio_) and the reaction rate constant (*k*_eq, abio_) for abiotic oxygen isotope exchange between NO_2_^−^ and H_2_O to be 11.9‰ and 1.13 × 10^−2^ h^−1^, respectively [45]. We assumed that ^18^ε_eq, AMX_ is identical to ^18^ε_eq, abio_ in the model simulation. Additionally, we determined three nitrogen isotope effects; (1) ^15^ε_NO2- → N2_, (2) ^15^ε_NO2- → NO3-_, and (6) ^15^ε_NH4 + − → NO2_ in this study.

Despite its significance, little information is available on the oxygen isotope effects (^18^ε) of individual anammox reaction pathways. Anammox, denitrification, and nitrification concurrently influence the oxygen isotope composition of nitrite (*δ*^18^O_NO2-_) and nitrate (*δ*^18^O_NO3-_), making the quantification of ^18^ε associated with individual anammox metabolism essential for estimating its contribution to *δ*^18^O_NO2-_ and *δ*^18^O_NO3-_ in the natural environments. This quantification is very complicated because three reactions influencing the values of *δ*^18^O_NO2_^−^ and *δ*^18^O_NO3_^−^ (NO₂^−^ reduction to N₂, NO₂^−^ oxidation to NO₃^−^, and NO₂^−^ equilibration with H₂O) occur simultaneously during anammox reaction, and the following four associated ^18^ε must be determined: (i) NO_2_^−^ reduction to N_2_ (^18^ε_NO2- → N2_), (ii) NO_2_^−^ oxidation to NO_3_^−^ (^18^ε_NO2- → NO3-_), (iii) incorporation of an O atom from H_2_O during NO_2_^−^ oxidation to NO_3_^−^ (^18^ε_H2O_), and (iv) abiotic and anammox-mediated oxygen isotope exchange between NO_2_^−^ and H_2_O (^18^ε_eq, abio_ and ^18^ε_eq, AMX_) (Fig. 1). To date, only the combined oxygen isotope effects for NO_2_^−^ oxidation to NO_3_^−^ (^18^E_NO2- → NO3-_ = 2/3 ^18^ε_NO2- → NO3-_ + 1/3 ^18^ε_H2O_) have been reported for three enrichment cultures of anammox bacteria [24]. Additionally, the oxygen isotope effect during the NO_2_^−^ oxidation to NO_3_^−^ (^18^ε_NO2- → NO3-_) has been estimated for biomass containing anammox bacteria from a wastewater treatment plant, but lacks high precision [25]. The individual oxygen isotope effects of each anammox reaction pathway (^18^ε_NO2- → N2,_  ^18^ε_NO2- → NO3-_ and ^18^ε_H2O_) remain entirely unexplored.

To interpret *δ*^18^O_NO2-_ and *δ*^18^O_NO3-_ in environments, it is essential to evaluate oxygen isotope uptake from ambient H_2_O and O_2_ into NO_2_^−^ and NO_3_^−^ during nitrification as well as abiotic or biologically enhanced oxygen isotope exchange between NO_2_^−^ and H_2_O. Oxygen isotope effects resulting from O atom uptake from O_2_ or H_2_O, along with O isotope exchange between NO_2_^−^ and H_2_O during nitrification, have been assessed by H_2_^18^O labeling experiments in various systems, including pure culture of ammonia-oxidizing bacteria (AOB) [27] and nitrite-oxidizing bacteria (NOB) [28], an enrichment culture of thermophilic ammonia-oxidizing archaea (AOA) [29], nitrifying cocultures and natural marine assemblages [30], and stream water [31]. Quantifying the influence of the O isotopic composition of water (*δ*^18^O_H2O_) is crucial, as *δ*^18^O_NO2-_ and *δ*^18^O_NO3-_ are strongly affected by both O isotope exchange between NO_2_^−^ and H_2_O and O atom uptake from H_2_O during nitrification.

Here we quantified the kinetic oxygen isotope effects (^18^ε_NO2- → N2,_  ^18^ε_NO2- → NO3-_ and ^18^ε_H2O_) as well as the rate constant for anammox-mediated O isotope exchange between NO_2_^−^ and H_2_O (*k*_*e*q, AMX_) associated with anammox metabolism in a marine anammox species “*Ca.* Scalindua sp.” [32]. We conducted a series of batch culture experiments with “*Ca*. Scalindua sp.” under varying *δ*^18^O_H2O_ medium conditions and determined the kinetic O isotope effects using a newly developed numerical model.

## Materials and methods

### Experimental design

The objective of this study was to determine the previously unexplored ^18^ε and O atom exchange between NO_2_^−^ and H_2_O associated with anammox metabolism using a highly enriched marine anammox culture of “*Ca.* Scalindua sp.”. To achieve this objective, we conducted a series of batch culture experiments with “*Ca*. Scalindua sp.” in different *δ*^18^O_H2O_ media and measured the time-dependent dynamics of concentrations and isotope compositions of nitrogen compounds (NH_4_^+^, NO_2_^−^, and NO_3_^−^). We estimated ^15^ε and ^18^ε for the respective reaction pathways of anammox metabolism using Markov Chain Monte Carlo (MCMC) method implemented in a newly constructed numerical model.

### Enrichment culture of “*Ca*. Scalindua sp.”

Free-living planktonic enrichment cultures of a marine anammox bacteria species “*Ca.* Scalindua sp.” were cultivated in a 3 L membrane bioreactor (MBR) equipped with a hollow fiber membrane module (pore size 0.1 μm, polyethylene) as previously described (Fig. S15) [33–35]. The inorganic nutrient medium continuously fed into the MBR contained the following: KH_2_PO_4_ (24.4 mg L^−1^), MgSO_4_·7H_2_O (99 mg L^−1^), CaCl_2_ (86 mg L^−1^), and 0.5 ml trace element solution I and II [36]. Equimolar amounts of NH_4_(SO_4_)_2_ and NaNO_2_ were added to achieve 10 mmol-N L^−1^. An artificial sea salt SEALIFE (Marine Tech, Tokyo, Japan) was supplemented into the media to achieve 2.5% salinity. The culture fluid in the MBR was continuously mixed with a magnetic stirrer at 200 rpm and sparged with 95% Ar-5% CO_2_ at a flow rate of 10 ml min^−1^. The pH was not controlled but remained between 7.3 and 7.8. The temperature was maintained at 30°C.

### Experiments for nitrogen and oxygen isotope effects during anammox

The enriched anammox bacterial cells were collected from MBRs operated at steady-state and further enriched by Percoll density gradient centrifugation [37]. The ratio of anammox bacteria to total cells (degree of enrichment) in the Percoll-centrifuged culture was >99% based on fluorescent *in situ* hybridization analysis. The highly enriched biomass was washed with mineral medium without NH_4_^+^ and NO_2_^−^ and resuspended in 0.9 L of the same medium (~10^9^ cells ml^−1^). The biomass was incubated in 1 L glass bottles (Shibata Scientific Technology, Ltd., Saitama, Japan) overnight to completely consume the residual NH_4_^+^ and NO_2_^−^. The media for batch culture experiments were prepared as described above, with the following modification: the *δ*^18^O of the H_2_O in the medium was adjusted to four different values (*δ*^18^O_H2O_ = −12.6‰ (unlabeled), 25.9‰, 56.7‰, and 110.1‰) by adding H_2_^18^O (97% ^18^O; Aldrich, prod. no. 329878). For each medium *δ*^18^O_H2O_ value, batch culture experiments were conducted in triplicate. The temperature was controlled at 30°C. The batch cultures were flushed with an Ar: CO_2_ gas mixture and stirred continuously. The pH was not controlled but remained between 7.2 and 7.9 (Average 7.5). The experiments were initiated by adding NaNO_2_ (at a final concentration of 2.0 mmol-N L^−1^) and (NH_4_)_2_SO_4_ (at a final concentration of 2.5 mmol L^−1^). The culture medium was continuously mixed using a magnetic stirrer at 200 rpm. To maintain anoxic conditions, a mixed gas (Ar: CO_2_ = 95:5) was purged into the culture medium at a flow rate of 10 m min^−1^. After adding substrates, a total of 25 to 40 ml of the culture solution was periodically sampled. Time course experiments lasted until the NO_2_^−^ had been completely consumed, which usually took approximately from 5 to 7 hours. All samples were immediately filtered using a 0.2-μm cellulose acetate filter (25CS020AN, Advantec, Tokyo, Japan). The pH was measured using 0.5 ml of the sample solution with a pH meter (pH meter B-712, Horiba, Ltd., Kyoto, Japan).

After filtration, sample solutions were immediately adjusted to pH 2 by adding 1 M H_2_SO_4_ solution and then stored at −20°C until analysis for nitrogen isotope ratios of NH_4_^+^ to prevent NH_4_^+^ from volatilizing. To analyse *δ*^15^N_NO2-_ and *δ*^18^O_NO2-_, sample solutions were immediately adjusted to pH 12 by adding 2 M low-N-blank NaOH solution after filtration and stored at −20°C until analysis to prevent oxygen isotope exchange between NO_2_^−^ and H_2_O during sample storage [38]. To analyse *δ*^15^N_NO3-_ and *δ*^18^O_NO3-_, any remaining NO_2_^−^ in the sample solution was immediately removed by adding sulphamic acid (H_3_NSO_3_) after filtration, as NO_2_^−^ interferes with NO_3_^−^ isotope analysis [39]. The concentration of NO_2_^−^ was measured using the naphthylethylenediamine method [40] to confirm complete removal of NO_2_^−^. The samples were then stored at −20°C until analysis.

### Experiments for nitrogen isotope exchange between NO_2_^−^ and NO_3_^−^

To investigate N isotope exchange between NO_2_^−^ and NO_3_^−^, batch culture experiments were conducted by adding 2 ml of 1 M (NH_4_)_2_SO_4_ solution, 2 ml of 0.75 M ACROS-NO_2_^−^ solution with a low *δ*^15^N value (*δ*^15^N_NO2-_ = −35.5‰), and 8 ml of 5 mM USGS32 (NO_3_^−^) solution with a high *δ*^15^N value (*δ*^15^N_NO3-_ = 180.0‰) to 800 ml culture medium. This experiment could only be performed once due to limited quantities of ACROS-NO_2_^−^ and USGS32 (NO_3_^−^) solutions. The final concentrations of NH_4_^+^, NO_2_^−^, and NO_3_^−^ were 2.5, 1.88, and 0.05 mM, respectively. Water with a *δ*^18^O of −12.6‰ was used as the medium. Other experimental procedures were the same as described above. In this experiment, the initial *δ*^15^N difference between NO_2_^−^ and NO_3_^−^ was set large (−215.5‰) to facilitate observation of rapid changes in *δ*^15^N_NO2−_ and *δ*^15^N_NO3-_ when N isotope exchange occurs during the anammox reaction.

### Chemical analyses

On the day of sampling, the following concentrations were analysed using 1.0 ml sample filtrate. The concentration of NH_4_^+^ was measured by the indophenol blue method [40] using a multi-label plate reader (ARVO MX 1420-01 J; PerkinElmer; Waltham, MA, USA). The NO_2_^−^ concentration was measured by the naphthylethylenediamine method [40]. The concentration of NO_3_^−^ was measured using ion chromatographs (IC-2010, TOSOH; Tokyo, Japan) equipped with a TSKgel IC-Anion HS column (TOSOH; Tokyo, Japan).

### Isotopic analyses

NH_4_^+^ nitrogen isotope analyses were performed using the ammonium diffusion method [41, 42] and subsequently measured by EA-IRMS (Flash EA1112, ConFlo IV interface, Delta plus Advantage; ThremoFinnigan). International and internal NH_4_^+^ isotopic standards, USGS25 (*δ*^15^N = −30.41‰), USGS26 (*δ*^15^N = 53.75‰), and IAEA-N-2 (*δ*^15^N = 20.3‰), were used for calibration. The one-sigma standard deviations of *δ*^15^N_NH_4_^+^_ measurements of standards were ± 0.3‰.

NO_2_^−^ nitrogen and oxygen isotope ratios were measured by chemically converting NO_2_^−^ to nitrous oxide (N_2_O) using the azide method [43]. All samples were adjusted to the same pH (pH = 12) and salinity (2.5% NaCl). The NO_2_^−^ standard solution had the same pH, salinity, solution volume and *δ*^18^O_H2O_ values as the batch of samples to account for any effects from pH-dependent incorporation of water O atoms into N_2_O during its generation. Due to the high sample pH (pH = 12), the azide buffer was modified by increasing the acetic acid concentration to 7.84 M [44] and the sodium azide concentration to 4 M [45]. The sample and standard solutions were buffered at pH 4.4 during the reaction. The N_2_O was then analysed in duplicate using a purge-and-trap, gas chromatography isotope ratio mass spectrometry (PT-GC-IRMS). The corrected *δ*^15^N and *δ*^18^O values of the sample N_2_O converted from NO_2_^−^ were calibrated against in-house NO_2_^−^ standards; JAM 1 (*δ*^15^N = −2.5‰, *δ*^18^O = 91.7‰), JAM 2 (*δ*^15^N = 1.8‰, *δ*^18^O = 9.9‰), JAM 3 (*δ*^15^N = −26.4‰, *δ*^18^O = 39.4‰), and JAM 4 (*δ*^15^N = −1.5‰, *δ*^18^O = −15.2‰) [45]. Each reference solution was analysed three times, and triplicate analyses generally yielded precisions of ±0.2‰ for *δ*^15^N_NO2-_ and ± 0.3‰ for *δ*^18^O_NO2-_. The details of sample preparation and measurement are described in our previous publication [45].

NO_3_^−^ nitrogen and oxygen isotope ratios were measured by microbial conversion of NO_3_^−^ to N_2_O using the denitrifier method [46, 47]. All samples were exactly adjusted to the same pH and salinity. The NO_3_^−^ standard solutions were prepared with the same pH, salinity and *δ*^18^O_H2O_ value as the batch of samples to be analysed. N_2_O was analysed in triplicate using PT-GC-IRMS. The corrected *δ*^15^N and *δ*^18^O values of the sample N_2_O converted from NO_3_^−^ were calibrated against international NO_3_^−^ isotopic standards: IAEAN3 (*δ*^15^N = 4.7‰, *δ*^18^O = 25.6‰), USGS32 (*δ*^15^N = 180‰, *δ*^18^O = 25.7‰), USGS34 (*δ*^15^N = −1.8‰, *δ*^18^O = −27.9‰), and USGS35 (*δ*^18^O = 57.5‰) [48]. Triplicate analyses generally yielded precisions of ±0.2‰ for *δ*^15^N_NO3-_ and ± 0.5‰ for *δ*^18^O_NO3-_. The details of sample preparation and measurement are described in our previous publication [45].

The *δ*^18^O_H2O_ was measured by equilibration with NO_2_^−^ and subsequent conversion of NO_2_^−^ to N_2_O using a modified azide method [49] with 0.5 ml of the samples and standards. The *δ*^18^O data was calibrated against Greenland Ice Sheet Precipitation (GISP, −24.8‰) and in-house water standards: Alaskan bottled mineral water (−19.0‰) and bottled de-salted seawater (0.2‰) (these *δ*^18^O values were determined by SI Science. Co. Ltd). Samples with high *δ*^18^O_H2O_ were mixed with MillQ water (−12.6‰) to yield measured *δ*^18^O values within the range of the standards (i.e. between −24.8‰ and 0.2‰), calculated according to the mixing ratio. Triplicate analyses yielded a precision of 0.20‰ for *δ*^18^O_H2O_.

### Calculation of isotopic effects with a numerical model

To estimate N and O isotope effects (^15^ε and ^18^ε) for the individual reaction pathways of anammox metabolism (Fig. 1), we developed and modified an ordinary differential equation model as described by Kotajima et al. (2020) [25] and Granger and Wankel (2016) [50] (see Supplemental Materials for details). The basic concept of our model involves taking mass balance of isotope pools (^14^N, ^15^N, ^16^O, and ^18^O) of NH_4_^+^, NO_2_^−^, and NO_3_^−^. The changes of each pool are expressed using differential equations. To infer each parameter on the N and O isotope effects, the differential equations were solved using the R package FME [51]. All simulations were run using this package, which provides solutions for differential equations with integration algorithms and inverse modeling using the Markov-Chain Monte-Carlo technique. Our model script in R language is available at ZENODO (the code is available at https://zenodo.org/records/15015868).

Because the anammox reaction was assumed to be a zero-order reaction in this model simulation, data points during the initial lag or low active phase were excluded for parameter estimations. The estimation range of each parameter related to N isotope effects was set as follows: (0 < ^15^ε_NH4 + →N2_ < 60, 0 < ^15^ε_NO2- → N2_ < 60, and − 60 < ^15^ε_NO2- → NO3-_ < 0), based on observed data sets and the reported ^15^ε values of anammox bacteria (Table S4) [23–25]. The MCMC were run for 100 000 iterations after a burn-in period of 50 000 iterations, and the posterior samples were obtained on the remaining iterations. We set the thinning rate was 5. The mean and standard deviation of isotope effect were derived by the overall posteriors consisted with the three independent replication. For the O isotope effects (^18^ε), uniform probabilities were used for each parameter, set as follows: (5 < ^18^ε_NO2- → N2_ < 20, −15 < ^18^ε_NO2- → NO3-_ < 0, 10 < ^18^ε_H2O_ < 25) (Table S5). These priors were based on observed data sets and reported ^18^ε values catalyzed by the same enzymes [28, 30, 31, 52]. The sampling number for the MCMC was also set to 100 000. Details of the model, calculation method and fitting results were described in the Supplementary Materials. To evaluate the validity of the calculation results from the model simulation, the N isotope effects of NH_4_^+^ oxidation (^15^ε_NH4 + →N2_), NO_2_^−^ reduction (^15^ε_NO2- → N2_) and NO_2_^−^ oxidation (^15^ε_NO2- → NO3-_) were also calculated by using the canonical closed-system Rayleigh isotope fractionation systematics [23].

## Results and discussion

### Batch culture experiments

**Figure 2:**
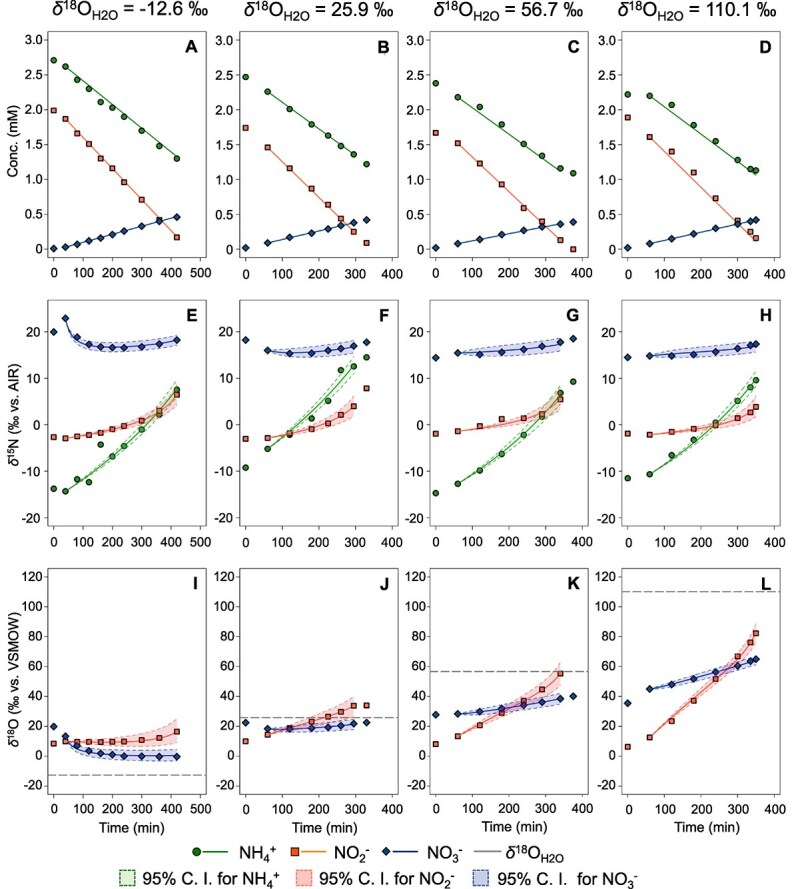
Changes in concentrations and nitrogen and oxygen isotope ratios of nitrogen compounds over time during anammox by “*Ca*. Scalindua sp.” in typical batch culture experiments with different *δ*^18^O_H2O_ of growth media. We adjusted the *δ*^18^O_H_2_O_ in the medium to four different values; −12.6‰ (A, E, I), 25.9‰ (B, F, J), 56.7‰ (C, G, K), and 110.1‰ (D, H, L), respectively. Concentrations (A - D), *δ*^15^N (E - H), and *δ*^18^O (I - L) of nitrogen compounds during the incubations. Symbols and lines represent experimental data and their model fitting by MCMC, respectively. 95% C.I. indicates 95% credible interval for estimated *δ*^15^N of NH_4_^+^, NO_2_^−^ and NO_3_^−^ (E - H) and *δ*^18^O of NO_2_^−^ and NO_3_^−^ (I - L). We performed all batch experiments in triplicate and presented only the results of the replication experiment 1. This is due to variations in sampling intervals, which depended on the activity of anammox biomass, making it difficult to merge the three-replicate data into a single figure using mean ± standard deviation. For other experimental results, please refer to the supplementary materials (Fig. S1-S4).

Batch culture experiments were performed in triplicate using four waters with different *δ*^18^O values (*δ*^18^O_H2O_ = −12.6‰ (unlabeled), 25.9‰, 56.7‰, and 110.1‰). The concentrations of NH_4_^+^, NO_2_^−^, and NO_3_^−^, and isotope ratios of nitrogen and oxygen (*δ*^15^N = ^15^N/^14^N and *δ*^18^O = ^18^O/^16^O) were measured over time during the batch culture. After addition of 2.5 mM of NH_4_^+^ and 2.0 mM of NO_2_^−^, both NH_4_^+^ and NO_2_^−^ were almost immediately consumed linearly with time, and NO_3_^−^ was concomitantly produced at constant rates (Fig. 2 A-D and Figs. S1-S4, A - C), indicating that the anammox reaction exhibited the maximum rates according to Michaelis–Menten kinetics at NO_2_^−^concentration ranges significantly higher than the half-saturation constants (*K_m_* for NO_2_^−^ ≈ 0.1–1.0 μM) for “*Ca*. Scalindua sp.” [53] (Supplementary materials Eq. S5-S7). In some experiments, short lag times and slightly lower active phases were observed (Fig. S2C, Fig. S3C and Fig. S4A). The average stoichiometric ratios of consumed NO_2_^−^ and consumed NH_4_^+^ (ΔNO_2_^−^/ΔNH_4_^+^) were 1.38 ± 0.10 (mean ± SD) and produced NO_3_^−^ and consumed NH_4_^+^ (ΔNO_3_^−^/ΔNH_4_^+^) were 0.33 ± 0.04, respectively. These values were slightly higher than those previously reported [2], but close to the one reported by Brunner et al. (2013) [23]. This may suggest that, in batch cultures, a greater proportion of NO_2_^−^ must be disproportionated to NO_3_^−^ and NO to facilitate NH_4_^+^ activation [54].

Nitrite was completely consumed within 295–450 min, whereas NH_4_^+^ remained in substantial amounts across all batch cultures (Fig. 2 A - D). Similar results were observed in three independent batch cultures (Figs. S1-S4 A - C), demonstrating the reproducibility of this study.

### *δ* ^15^N dynamics of nitrogen compounds

*δ* ^15^N_NH4+_ steadily increased during the anammox reaction, indicating preferential consumption of the ^14^N isotope of NH_4_^+^ i.e. normal kinetic isotope effect (Fig. 2 E-H and Figs. S1-S4 D - F). *δ*^15^N_NO2-_ increased exponentially at the end of experiments, whereas *δ*^15^N_NO3-_ decreased slightly at the beginning and increased to larger values at the end of all experiments. *δ*^15^N_NO2-_ was affected by both NO_2_^−^ reduction to N_2_ and NO_2_^−^ oxidation to NO_3_^−^ (Fig. 1). The *δ*^15^N of the newly produced NO_3_^−^ was much higher than *δ*^15^N_NO2-_ in all experiments, indicating that the ^15^N isotope of NO_2_^−^ was preferentially incorporated into NO_3_^−^, i.e. the inverse isotope effect [25].

Anammox bacteria oxidize NO_2_^−^ to NO_3_^−^ and also can reverse this enzymatic reaction [55, 56]. A previous study demonstrated that N isotope exchange between NO_2_^−^ and NO_3_^−^ (^15^ε_NO2-↔︎NO3-_ = −60.5‰) occurred rapidly during the early growth phase [23]; however, this phenomenon was not consistently observed in their study or in our previous study [24]. To investigate N isotope exchange between NO_2_^−^ and NO_3_^−^, we conducted a batch culture experiment using ^15^N-poor NO_2_^−^ (ACROS; *δ*^15^N_NO2-_ = −35.5‰) and ^15^N-rich NO_3_^−^ (USGS32; *δ*^15^N_NO3_- = 180‰), along with unlabeled water (*δ*^18^O_H2O_ = −12.6‰). Given the large initial *δ*^15^N difference between NO_2_^−^ and NO_3_^−^ (−215.5‰), any occurrence of N isotope exchange would be expected to induce an abrupt change in *δ*^15^N_NO2-_ and *δ*^15^N_NO3-_ during the anammox reaction.

**Figure 3:**
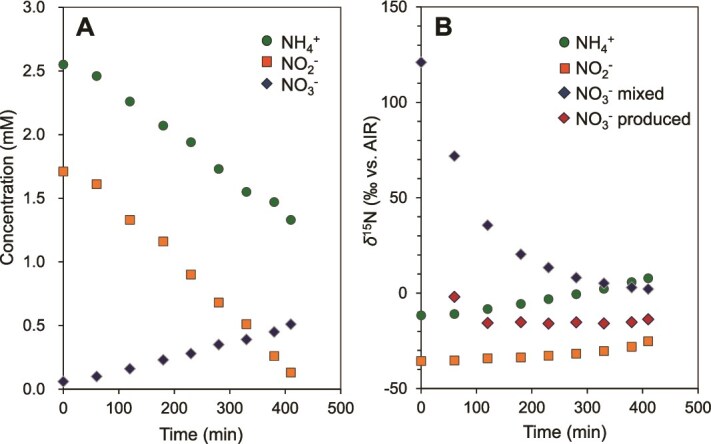
Changes in concentrations (A) and *δ*^15^N values of nitrogen compounds (B) over time during anammox batch culture experiments with ^15^N-poor nitrite (ACROS; *δ*^15^N_NO2-_ = −35.5‰) and ^15^N-rich nitrate (USGS32; *δ*^15^N_NO3_- = 180‰). In panel B, the *δ*^15^N_NO3- mixed_ represents the measured *δ*^15^N value of mixed NO_3_^−^ (USGS32 and newly produced NO_3_^−^), and the *δ*^15^N_NO3- produced_ represents the calculated *δ*^15^N value of newly produced NO_3_^−^ using a following equation: *δ*^15^N_NO3- produced_ = (C _t = t_ × *δ*^15^N_NO3- t = t_ - C _t = 0_ × *δ*^15^N_NO3- t = 0_) / (C _t = t_ – C _t = 0_). According to this equation, the *δ*^15^N_NO3- produced_ at t = 0 cannot be plotted because it is calculated from the difference from the initial value.

The simultaneous consumption of NH_4_^+^ and NO_2_^−^, along with the formation of NO_3_^−^, was observed, aligning with the previously reported anammox stoichiometry (Fig. 3A). Approximately 0.45 mM of NO_3_^−^ was newly produced from ^15^N-poor NO_2_^−^. At t = 0, *δ*^15^N_NO3-_ was lower than the original N isotopic ratio of USGS32 (180‰) and subsequently decreased asymptotically over time as NO_3_^−^ was generated from^15^N-poor NO_2_^−^ (Fig. 3B). No abrupt changes in *δ*^15^N_NO2-_ or *δ*^15^N_NO3-_ were observed throughout the anammox reaction. The N isotope ratio of the newly produced NO_3_^−^ (*δ*^15^N_NO3- produced_) was calculated using the following equation.

*δ* ^15^N_NO3- produced_ = (C _t = t_ × *δ*^15^N_NO3- t = t_ - C _t = 0_ × *δ*^15^N_NO3- t = 0_) / (C _t = t_ – C _t = 0_).

The background NO_3_^−^ concentration and *δ*^15^N_NO3-_ in the artificial seawater were 0.02 mM and 20‰, respectively. After mixing 800 ml of artificial seawater with 8 ml of USGS32 (5 mM NO_3_^−^, *δ*^15^N = 180‰), the NO_3_^−^ concentration and *δ*^15^N of mixed NO_3_^−^ at t = 0 were measured as 0.06 mM and 126‰, respectively. These values were in close agreement with the calculated estimates. Thus, the initial decrease in *δ*^15^N_NO3- produced_ is likely due to dilution by NO_3_^−^ present in the artificial seawater (Fig. 3B). In addition, NO_3_^−^ production is minimal at the early stage of batch culture, making *δ*^15^N_NO3- produced_ highly susceptible to errors in concentration and N isotope measurements.

Nitrogen isotope exchange between NO_2_^−^ and NO_3_^−^ was observed only during the initial, low-activity growth phase but was negligible in the later phase when anammox activity was fully established [23]. In all batch experiments conducted in this study, active and stoichiometrically balanced anammox reactions were initiated without distinct lag phases. Consequently, no significant N isotope exchange between NO_2_^−^ and NO_3_^−^ was observed, and it was therefore excluded from the subsequent numerical model simulation used to estimate nitrogen and oxygen isotope effects.

### Nitrogen isotope effects (^15^ε)

The kinetic nitrogen isotope effects (^15^ε) for each reaction pathway were estimated using a numerical model based on Bayesian estimation, implemented with a MCMC algorithm (Fig. 1, Table 1A, Table S1A). The model simulations achieved highly successful curve-fitting across all experiments (Fig. 2 E - H and Figs. S1-S4 D - F). The mean value of ^15^ε_NH4 + →N2_ was 30.9 ± 3.3‰ (mean ± SD), in good agreement with the values obtained from continuous culture experiments of “*Ca.* Scalindua sp.” (^15^ε_NH4 + →N2_ = 32.7 ± 0.7‰) [24] and the value from batch culture experiments of “*Ca.* Kuenenia stuttgartiensis” (^15^ε _NH4 + →N2_ = 23.5 ~ 29.1‰) [23]. The mean values of ^15^ε_NO2- → N2_ and ^15^ε_NO2- → NO3_^−^ were 9.7 ± 1.7‰ and − 17.3 ± 1.2‰, respectively. These values are smaller than the reported values of “*Ca*. Scalindua sp.” (^15^ε_NO2- → N2_ = 19.9 ± 1.7‰ and ^15^ε_NO2- → NO3-_ = −30.1 ± 3.0‰) [24]. This difference may have resulted from differences in growth conditions (water used for the culture medium, the concentrations of NH_4_^+^ and NO_2_^−^, and anammox activity) and cultivation methods (batch culture vs. continuous culture).

**Table 1:** Summary of nitrogen (**A**) and oxygen isotope effects and a reaction rate constant (*k*_eq, AMX_) for anammox-mediated oxygen isotope exchange between NO_2_^−^ and H_2_O (**B**) during anammox metabolism determined from batch culture experiments with different *δ*^18^O_H2O_ media using a newly developed numerical model implemented with a MCMC algorithm.

**A**					
*δ* ^18^O_H2O_ of medium (‰)		n	^15^ε_AMO_	^15^ε_NIR_	^15^ε_NXR_
			^15^ε_NH4+ → N2_ (‰)	^15^ε_NO2- → N2_ (‰)	^15^ε_NO2- → NO3-_ (‰)
−12.6		3	30.2 ± 3.1	11.7 ± 0.6	−16.9 ± 1.0
25.9		3	33.7 ± 3.0	10.2 ± 1.1	−17.0 ± 1.0
56.7		3	28.9 ± 1.8	8.4 ± 1.3	−17.6 ± 1.4
110.1		3	30.9 ± 3.0	8.5 ± 0.7	−17.9 ± 1.0
Average		12	30.9 ± 3.3	9.7 ± 1.7	−17.3 ± 1.2
**B**					
*δ* ^18^O_H2O_ of medium (‰)	n	^18^ε_NIR_	^18^ε_NXR_		
		^18^ε_NO2- → N2_ ‰	^18^ε_NO2- → NO3-_ (‰)	^18^ε_H2O_ (‰)	*k*_eq, AMX_ (×10^−2^ h^−1^)
−12.6	3	10.6 ± 2.5	−2.9 ± 1.9	19.2 ± 3.8	13.56 ± 5.38
25.9	3	12.1 ± 2.5	−6.3 ± 2.5	17.7 ± 4.3	8.44 ± 4.63
56.7	3	13.8 ± 2.6	−9.8 ± 2.5	17.3 ± 4.2	11.28 ± 2.54
110.1	3	16.1 ± 3.2	−11.0 ± 2.1	16.4 ± 4.0	12.44 ± 3.13

The values are the overall mean and SD of the three posteriors obtained by each batch experiment replicate.

To assess the validity of the model simulation results, we also determined the kinetic nitrogen isotope effects of NH_4_^+^ oxidation, NO_2_^−^ reduction, and NO_2_^−^ oxidation using a closed-system Rayleigh model, as previously described [23] (Table S2, Figs. S5-S7). The calculated ^15^ε values for NH_4_^+^ oxidation and NO_2_^−^ oxidation (^15^ε_NH4 + →N2_ = 27.5‰ ~ 33.3‰ and ^15^ε_NO2- → NO3-_ = −17.7‰ ~ −19.0‰) (Table S2) closely matched those obtained from our model fitting (Table 1A), whereas ^15^ε_NO2- → N2_ ranged from 0.5‰ to 2.4‰ (*δ*^15^N_NO2-_ based) and from 1.5‰ to 8.6‰ (*δ*^15^N_NO3-_ based) (Table S2), which were slightly lower than the values obtained from the model fitting (8.4‰ ~ 11.7‰; Table 1A). A similar discrepancy in ^15^ε estimations has been reported previously [23], however, the exact reason is currently unknown.

### *δ* ^18^O dynamics of nitrogen compounds

**Figure 4:**
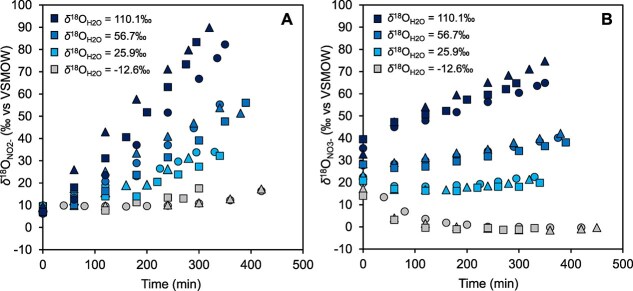
Summary of changes in *δ*^18^O values of NO_2_^−^ (A) and NO_3_^−^ (B) over time during anammox batch culture experiments with different *δ*^18^O_H2O_ of growth media (−12.6‰, 25.9‰, 56.7‰, and 110.1‰). We conducted the batch culture experiments in triplicates for each *δ*^18^O_H2O_ media (1^st^: Circle, 2^nd^: Triangle, 3^rd^: Square).

During the anammox reaction, *δ*^18^O_NO2-_ values rapidly approached equilibrium between NO_2_^−^ and H_2_O within 6–7 hours of incubation in high *δ*^18^O_H2O_ media (Fig. 4A and Fig. S8). In contrast, in abiotic conditions, the equilibrium was achieved within ca. 650 h (Fig. S8) [45]. This indicates that “*Ca.* Scalindua sp.” significantly promoted O isotope exchange between NO_2_^−^ and H_2_O. This is probably due to three reasons: (i) the lower pH in anammoxosome, (ii) the reversibility of NO_2_^−^ oxidation reaction, and (iii) the reversibility of NO_2_^−^ reduction reaction, as explained in detail below. First, an anammox reaction occurs within anammoxosome, the pH of which is known to be around 6 [57], more than one unit lower than the culture medium (pH ≈ 7.5). The oxygen exchange rate has been reported to increase as the pH decreases [22]. At 30°C, the rate of abiotic oxygen exchange *k*_eq_ was estimated to be 4.39 × 10^−2^ h^−1^ at pH 6 and 1.13 × 10^−2^ h^−1^ at pH 7.5 [22]. These values indicate considerable differences in the O isotope exchange between NO_2_^−^ and H_2_O within the anammoxosome. Second, when NO_2_^−^ is oxidized to NO_3_^−^, first a complex of the enzyme nitrite oxidoreductase and O atom derived from H_2_O is formed, then this enzyme-oxygen complex combines with NO_2_^−^ to form a transition state [28, 58]. This transition state (temporarily containing three O atoms) can either cleave the enzyme to form NO_3_^−^ or lose one of the three O atoms and decompose back to NO_2_ [28, 58]. If the original O atom of NO_2_^−^ is lost during the reverse reaction, it is replaced by an O atom of H_2_O. Third, in the NO_2_^−^ reduction reaction by “*Ca*. Scalindua sp.”, NO_2_^−^ is first reduced to nitric oxide (NO), which is then combined with NH_4_^+^ and converted to hydrazine (N_2_H_4_) [59]. Because the latter hydrazine synthesis is the rate-limiting step [1], NO may accumulate slightly in the anammoxozome [60] and some of which may acquire O atoms from H_2_O and be re-oxidized back to NO_2_^−^. This reverse reaction would further promote O isotope exchange between NO_2_^−^ and H_2_O. Similar microbially facilitated O isotope exchange between NO_2_^−^ and H_2_O has also been reported in aerobic AOB [27] and archaea (AOA) [29], as well as nitrite-oxidizing bacteria (NOB) [28, 30, 31].

The *δ*^18^O_NO3-_ values appeared to depend on the *δ*^18^O_H2O_ media (Fig. 4B). When the *δ*^18^O_H2O_ media value was −12.6‰, the *δ*^18^O_NO3-_ decreased from 15‰ to −0.3‰. Similarly, at *δ*^18^O_H2O_ values of 25.9‰, 56.7‰, and 110.1‰, the final *δ*^18^O_NO3-_ values were approximately 22‰, 42‰, and 64‰, respectively. These results indicate that the *δ*^18^O_NO3-, produced_ are gradually approaches equilibrium with the *δ*^18^O_H2O_ medium by incorporating O atoms from water, although full equilibrium has not yet been reached. The change in *δ*^18^O_NO2-_ occurred more rapid than that in *δ*^18^O_NO3-, produced_ (Fig. S9), suggesting that O isotope exchange between NO_2_^−^ and H_2_O played a significant role.

### Dependence of *δ*^18^O_NO2-_ and *δ*^18^O_NO3-_ on *δ*^18^O_H2O_

**Figure 5:**
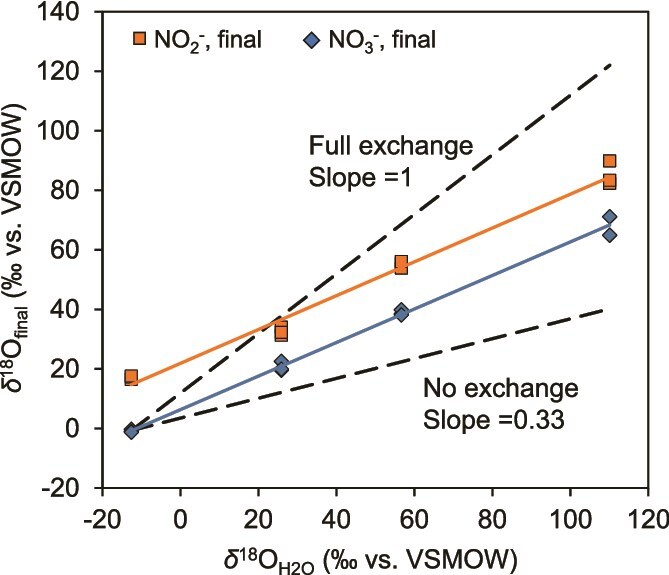
Relationship between final values of *δ*^18^O_NO2-_ and *δ*^18^O_NO3-_ and *δ*^18^O_H2O_ media. The final values of *δ*^18^O_NO2-, final_ and *δ*^18^O_NO3-, final_ at the end of incubation (when NO_2_^−^ was almost consumed) were plotted against *δ*^18^O_H2O_. All triplicate data were plotted in this figure. The dashed black lines indicate estimated full exchange (slope = 1, 100% incorporation) and no exchange (33% incorporation) for NO_3_^−^. The slopes of the regression lines of *δ*^18^O_NO2-, final_ and *δ*^18^O_NO3-, final_ were 0.568 and 0.563, respectively, indicating that 34% of O atoms in NO_2_^−^ were exchanged with H_2_O before oxidation to NO₃^−^ (see supplementary materials for details of this calculation).

The values of *δ*^18^O_NO2-, final_ and *δ*^18^O_NO3-, final_ at the end of incubation (when NO_2_^−^ was almost consumed) were plotted against *δ*^18^O_H2O_, and both showed the same linear relationship with the same slope of 0.56 (Fig. 5). According to the definition of *δ*^18^O_NO3-, final_ [22, 27], this indicates that 34% of O atoms in NO_2_^−^ were exchanged with H_2_O before oxidation to NO₃^−^ (see Supplementary materials for details of this calculation). Additionally, one O atom was incorporated from H_2_O into NO_2_^−^ during its oxidation to NO_3_^−^ [27, 61]. These findings suggest that, depending on the extent of equilibration, all three O atoms in the resulting NO₃^−^ may carry the isotopic signature of water. As a result, both *δ*^18^O_NO2-_ and *δ*^18^O_NO3-_ in the anammox reaction were strongly dependent on *δ*^18^O_H2O_, even though their oxygen isotopic systematics are different: *δ*^18^O_NO3-_ is influenced by isotopic fractionation of NO_2_^−^ oxidation and O uptake from H_2_O during NO_2_^−^ oxidation, whereas *δ*^18^O_NO2-_ is influenced by isotopic fractionations of NO_2_^−^ oxidation, NO_2_^−^ reduction, and O isotope exchange between NO_2_^−^ and H_2_O (Fig. 1). The specific mechanism underlying why *δ*^18^O_NO2-, final_ and *δ*^18^O_NO3-, final_ exhibit the same correlation (slope) with *δ*^18^O_H2O_ remains unknown at this time.

Based on these observations, we incorporated the reaction rate constants (*k*_eq, abio_ and *k*_eq, AMX_) and equilibrium isotope effects (^18^ε_eq, abio_ and ^18^ε_eq, AMX_) of both abiotic and anammox-mediated O isotope exchange processes into the numerical model to simulate O isotope exchange between NO_2_^−^ and H_2_O via anammox (Fig. 1). To determine *k*_eq, AMX_ and ^18^ε_eq, AMX_ using the numerical model, we assumed that ^18^ε_eq, AMX_ was identical to the abiotic equilibrium isotope effect (^18^ε_eq, abio_ = 11.9‰) [45]. This assumption is based on the principle that an enzyme does not alter the equilibrium point but can accelerate the rate at which equilibrium is reached [28].

### Numerical model simulation

A closed-system Rayleigh model is not applicable to the oxygen isotope systematics of anammox, as multiple reactions including O isotope exchange between NO_2_^−^ and H_2_O influence the oxygen isotopic composition of NO_2_^−^ and NO_3_^−^ (Fig. 1). The Rayleigh model is designed for unidirectional reactions with a single product and relies on certain approximations to maintain linearity. Therefore, the oxygen isotope effects (^18^*ε*) during anammox were directly estimated using a Bayesian approach in a numerical model implemented with a MCMC algorithm. The MCMC algorithm is a powerful tool capable of estimating multiple parameters and their uncertainties through iterative random sampling. This method does not require any approximations and enables the validation of various types of models. The formulation of the nitrogen and oxygen isotopic model of the anammox reaction is described in the Supplementary Materials.

We estimated three kinetic O isotope effects (^18^ε_NO2- → N2,_  ^18^ε_NO2- → NO3-_ and ^18^ε_H2O_) as well as the rate constant for O isotope exchange between NO_2_^−^ and H_2_O (*k*_*e*q, AMX_) associated with anammox reaction using the numerical model with default parameter settings (Table S4). The curve-fitting results, along with 95% confidence intervals for temporal changes in concentrations and oxygen isotopic ratios of nitrogen compounds in each batch experiment, are presented in Figs. 2 I - L and Figs. S1-S4 G - I.

### Oxygen isotope effects (^18^ε)

**Figure 6:**
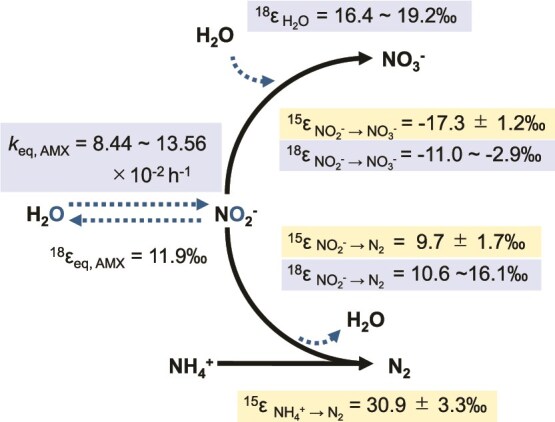
Summary of N and O isotope effects associated with anammox. These N and O isotope effects were determined at different *δ*^18^O_H2O_ of growth media (−12.6‰, 25.9‰, 56.7‰, and 110.1‰) in this study. The values of nitrogen isotope effects are overall mean and SD of twelve posteriors (including different *δ*^18^O_H2O_ of growth media) obtained from a numerical model implemented with a MCMC algorithm. The values of oxygen isotope effects are the ranges of overall means of three posteriors based on different *δ*^18^O_H2O_ of growth media. The equilibrium isotope effect (^18^ε_eq, AMX_) for anammox-mediated oxygen isotope exchange between NO_2_^−^ and H_2_O was assumed to be identical to ^18^ε_eq, abio_ (11.9‰), as previously determined [45].

The estimated oxygen isotope effects (^18^ε) and reaction rate constants for anammox-mediated O isotope exchange between NO_2_^−^ and H_2_O (*k*_eq, AMX_) are summarized in Fig. 6, Table 1B and Table S1B. The overall posterior means ± SD of *k*_eq, AMX_ determined by 100 000 iterations with the MCMC algorithm were 13.56 ± 5.38, 8.44 ± 4.63, 11.28 ± 2.54 and 12.44 ± 3.13 (× 10^−2^ h^−1^) at *δ*^18^O_H2O_ of −12.6‰, 25.9‰, 56.7‰ and 110.1‰, respectively (Table 1B). These *k*_eq, AMX_ values were 7.5 to 12 times higher than the reaction rate constant of abiotic O isotope exchange (*k*_eq, abio_ = 1.13 × 10^−2^ h^−1^) [45]. The kinetic O isotope effects during NO_2_^−^ reduction to N_2_, ^18^ε_NO2- → N2_, were 10.6 ± 2.5‰, 12.1 ± 2.5‰, 13.8 ± 2.6‰ and 16.1 ± 3.2‰ at *δ*^18^O_H2O_ of −12.6‰, 25.9‰, 56.7‰ and 110.1‰, respectively (Table 1B). The O isotope effects of NO_2_^−^ reduction catalyzed by copper-containing nitrite reductase (Cu-NIR) and cytochrome *cd_1_*-containing nitrite reductase (Fe-NIR) (both catalyse the reduction of NO_2_^−^ to NO) were determined for several denitrifying bacterial strains [52]. Denitrifier strains with Fe-NIR exhibited a slightly larger O isotope effect (^18^ε_NO2- → N2_ = 6 ± 2‰) than denitrifier strains with Cu-NIR (^18^ε_NO2- → N2_ = 2 ± 2‰) [52]. “*Ca.* Scalindua sp.” showed larger O isotope effects (^18^ε_NO2- → N2_ = 10.6 to 16.1‰) than denitrifier strains with Fe-NIR [52]. The identity and function of nitrite reductase in “*Ca*. Scalindua sp.” remain under debate [62], and further research is needed to elucidate its details.

For NO_2_^−^ oxidation to NO_3_^−^, the inverse isotope effect (^18^ε_NO2- → NO3-_) was determined as −2.9 ± 1.9‰, −6.3 ± 2.5‰, −9.8 ± 2.5‰ and − 11.0 ± 2.1‰ at *δ*^18^O_H2O_ values of −12.6‰, 25.9‰, 56.7‰, and 110.1‰, respectively (Table 1B). Nitrite-oxidizing bacteria (NOB) also exhibited inverse kinetic O isotope effects (^18^ε_NO2- → NO3-_ = −1.3 ± 0.4‰ to −8.2 ± 2.6‰) [28]. The kinetic O isotope effect associated with the incorporation of O atom from H_2_O during NO_2_^−^ oxidation to NO_3_^−^, ^18^ε_H2O_, was 19.2 ± 3.8‰, 17.7 ± 4.3‰, 17.3 ± 4.2‰, and 16.4 ± 4.0‰ when *δ*^18^O_H2O_ was 12.6‰, 25.9‰, 56.7‰, and 110.1‰, respectively (Table 1B). The ^18^ε_H2O_ values were positive, indicating a normal kinetic O isotope effect, similar to those observed in NOB [28]. H_2_^16^O were preferentially incorporated into NO_3_^−^, resulting in the *δ*^18^O_NO3-_ values lower than the *δ*^18^O_NO2-_ values at the end of all anammox batch experiments (Figs. 2 I - L and Figs. S1-S4 G - I).

### Evaluation of estimated parameters

The estimated parameters and their 95% confidence interval are summarized in Fig. S10. The variability in the estimated O isotope effects may be attributed to the fact that *δ*^18^O_NO2-_ and *δ*^18^O_NO3-_ converged with the *δ*^18^O_H2O_ at rates significantly faster than currently expected (Fig. 4 and Fig. S8). This finding suggests the presence of unknown pathways or processes facilitating O atom exchange between H_2_O and NO_2_^−^. To characterize the unexpectedly rapid anammox-mediated O isotope exchange between NO_2_^−^ and H_2_O, we introduced a reaction rate constant (*k*_eq, AMX_). Pairs plot analyses revealed a linear correlation between *k*_eq, AMX_ and ^18^ε_NO2- → N2_ (Fig. S11). To assess the influence of *k*_eq, AMX_ on the estimation of other parameters (^18^ε_NO2- → N2_, ^18^ε_NO2- → NO3-_, and ^18^ε_H2O_), model simulations were performed with *k*_eq, AMX_ values fixed at 10.8, 12.0, 13.2, 15.0, or 18.0 (×10^−2^ h^−1^) (Fig. S12). When *k*_eq, AMX_ was fixed at 15.0 × 10^−2^ h^−1^, the narrowest ranges, showing minimal dependence on *δ*^18^O_H2O_, were obtained for ^18^ε_NO2- → N2_ (11.2 ± 1.2‰, 7.8 ± 1.6‰, 9.3 ± 1.7‰ and 11.2 ± 3.5‰) and ^18^ε_H2O_ (19.0 ± 3.9‰, 18.0 ± 4.3‰, 17.1 ± 4.3‰ and 17.5 ± 4.3‰) at *δ*^18^O_H2O_ values of −12.6‰, 25.9‰, 56.7‰, and 110.1‰, respectively (Fig. S12 A and C), whereas the variations in *k*_eq, AMX_ did not affect ^18^ε_NO2- → NO3-_ (Fig. S12B). To evaluate the impact of ^18^ε_NO2- → NO3-_ on the curve-fitting results for *δ*^18^O_NO2-_ and *δ*^18^O_NO3-_, model simulations were conducted with ^18^ε_NO2- → NO3-_ values fixed at 0‰, −5.0‰ and − 9.7‰ (Fig. S13). As ^18^ε_NO2- → NO3-_ became more negative, *δ*^18^O_NO3-_ increased progressively, achieving optimal curve-fitting at ^18^ε_NO2- → NO3-_ value of −9.7‰. In batch experiments with higher *δ*^18^O_H2O_ values, the stronger negative ^18^ε_NO2- → NO3-_ values may serve to compensate for the observed increase in *δ*^18^O_NO3-_.

Another possible explanation for the convergence of *δ*^18^O_NO2-_ and *δ*^18^O_NO3-_ values toward *δ*^18^O_H2O_ is the reverse reaction of NO_2_^−^ oxidation (i.e. NO_3_^−^ reduction or a NO_2_^−^ transition state decomposes back to NO_2_^−^) facilitated by the enzyme nitrite oxidoreductase (NXR). To assess the effect of NO_2_^−^ oxidation reversibility on curve-fitting, we incorporated the backward flux of NO_2_^−^ oxidation (i.e. NO_3_^−^ reduction) into the model simulation. Model simulations were conducted using the ratio of NO_3_^−^ reduction rate (backward flux) to NO_2_^−^ oxidation rate (forward flux) (Fig. S14). Even under conditions assuming large backward fluxes (up to 75%), the rapid increases in *δ*^18^O_NO3-_ and *δ*^18^O_NO2-_ over time, particularly the convergence of *δ*^18^O_NO2-_ with *δ*^18^O_NO2-, eq_, could not be reproduced. The simulated reversibility (up to 75%) was significantly greater than previously observed reverse reactions mediated by NXR in anammox bacteria [63]. This discrepancy is likely due to the relatively smaller reaction pool of NO_2_^−^ oxidation to NO_3_^−^ compared to NO_2_^−^ reduction to N_2_ in the anammox metabolism. Because the reversibility of NO₂^−^ oxidation has a limited effect on *δ*^18^O_NO₂_^−^, excluding the backward flux of NO₂^−^ oxidation from parameter estimation in the model simulations is a reasonable approach.

Oxygen isotope exchange between NO_2_^−^ and H_2_O associated with anammox can diminish or overwrite the isotope signals of NO_2_^−^ reduction and oxidation, yielding NO_3_^−^ with *δ*^18^O values closely aligned with those of ambient water. A similar influence of *δ*^18^O_H2O_ on *δ*^18^O_NO3-_ was observed in nitrification [31]. The *δ*^18^O values of NO_3_^−^ produced via NO_2_^−^ oxidation could not be explained solely by a 2: 1 incorporation ratio of water and molecular O atoms [31]. Instead, these values were largely modulated by O exchange between H_2_O and NO_2_^−^, as well as kinetic and equilibrium isotope effects influencing O atom incorporation from both sources [31]. Consequently, *δ*^18^O_NO3-_ generally converges with *δ*^18^O_H2O_, and the reported *δ*^18^O_NO3-_ values may not necessarily reflect the isotope signature of nitrification [31]. Anoxic incubation experiments using natural sediments containing both NO_2_^—^oxidizing and denitrifying microorganisms revealed that *δ*^18^O_NO3-_ was significantly affected by the extent of O atom incorporation from ambient H_2_O [64]. This suggests that O atom incorporation into NO_3_^−^ by NO_2_^—^oxidizing microorganism can override the original O isotope signature of denitrification [64].

### Influence of *δ*^18^O_H2O_ on NO_3_^−^ Δ*δ*^18^O: Δ*δ*^15^N trajectory during anammox reaction (model simulation excises)

NO_3_^−^ is the primary bioavailable form of nitrogen in the ocean, and its natural abundance and stable isotope ratios of N and O (*δ*^15^N_NO3-_ and *δ*^18^O_NO3-_) serve as valuable markers for tracking the biogeochemical conversion processes of NO_3_^−^ in the natural environment. Early studies suggested that the heavy O and N isotopes in NO_3_^−^ are proportionally enriched during denitrification and that the N and O isotopic effects of NO_3_^−^ are nearly identical (i.e. ^18^ε: ^15^ε = 1) [65–67]. Deviations from the expected trajectory of NO_3_^−^ Δ*δ*^18^O: Δ*δ*^15^N = 1 have been attributed to the inverse kinetic isotope effect associated with canonical nitrite oxidation [14, 50, 67].

To evaluate the impact of *δ*^18^O_H2O_ on the trajectories of Δ*δ*^18^O_NO3-_: Δ*δ*^15^N_NO3-_ for anammox, model simulations were performed using the N and O isotope effects determined in this study, with *δ*^18^O_H2O_ values ranging from −7.7‰ to 1.8‰ (refer to Supplementary Materials text for details on the parameter setting). It was assumed that complete isotopic equilibrium had been established between NO₂^−^ and H₂O. All the parameters used in the model simulation exercises are provided in Table S5.

**Figure 7:**
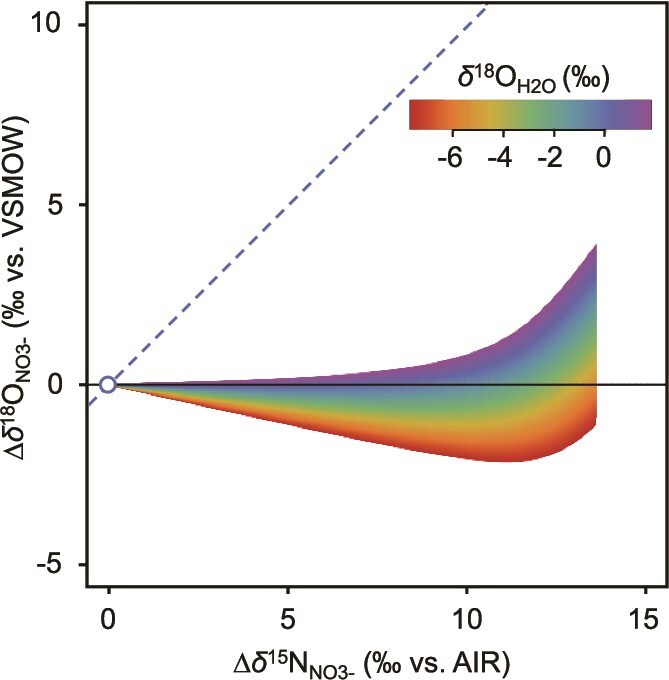
Influence of *δ*^18^O_H2O_ on Δ*δ*^18^O_NO3-_: Δ*δ*^15^N_NO3-_ as predicted by model simulation. Model simulations were performed using *δ*^*1*8^O_H₂O_ values ranging from −7.7‰ to 1.8‰, with parameters provided in Table S5. Complete isotopic equilibrium between NO₂^−^ and H₂O was assumed. Δ*δ*^18^O_NO3-_ (*δ*^18^O_NO3-_ - *δ*^18^O_NO3-, initial_) was plotted against the corresponding Δ*δ*^15^N_NO3-_ (*δ*^15^N_NO3-_ - *δ*^15^N_NO3-, initial_) for varying *δ*^18^O_H2O_ values. The dotted line represents the trajectory of Δ*δ*^18^O_NO3-_: Δ*δ*^15^N_NO3-_ = 1.

Model simulation revealed that *δ*^18^O_H2O_ significantly influences the trajectories of Δ*δ*^18^O_NO3-_: Δ*δ*^15^N_NO3-_ even within a relatively small range of *δ*^18^O_H2O_ values (*δ*^18^O_H2O_ = −7.7‰ to 1.8‰) (Fig. 7). Under these simulation settings (Table S5), the *δ*^18^O value of NO_3_^−^ produced from NO_2_^−^ oxidation via anammox is directly linked to *δ*^18^O_H2O_ (Fig. 3 and Fig. S9). Consequently, the trajectory of NO_3_^−^ Δ*δ*^18^O: Δ*δ*^15^N remained below 0.3, regardless of *δ*^18^O_H2O_, due to lower *δ*^18^O_NO3-_ values (Fig. 7). This suggests that the inverse kinetic N and O isotope effects associated with anammox-driven NO_2_^−^ oxidation, coupled with the rapid exchange of O atom between NO_2_^−^ and H_2_O, result in a deviation from the expected trajectory of NO_3_^−^ Δ*δ*^18^O: Δ*δ*^15^N = 1, similar to that observed in canonical nitrite oxidation.

In microbial NO_3_^−^ reduction systems, several studies have reported that NO_3_^−^ reduction mediated by Nap (periplasmic nitrate reductase) increases the *δ*^15^N of residual NO_3_^−^ relative to *δ*^18^O, resulting in a trajectory of NO_3_^−^ Δ*δ*^18^O: Δ*δ*^15^N ≈ 0.5 [65, 67–69]. Conversely, a recent study found that the trajectory of NO_3_^−^ Δ*δ*^18^O: Δ*δ*^15^N remained stable at ~1, even in denitrifying enrichment cultures with varying levels of NapA and NarG expression [70]. These contrasting findings indicate that uncertainties remain in quantifying the contributions of NO_2_^−^ oxidation by anammox and NO_3_^−^ reduction by Nap based solely on the NO_3_^−^ Δ*δ*^18^O: Δ*δ*^15^N, emphasizing the need for further research.

In conclusion, because “*Ca.* Scalindua sp.” significantly enhanced oxygen isotope exchange between NO_2_^−^ and H_2_O, we proposed a new rate constant for anammox-mediated oxygen isotope exchange (*k*_eq, AMX_ = 8.44 ~ 13.56 × 10^−2^ h^−1^), incorporated it into a newly developed numerical model, and determined the previously unexplored oxygen isotope effects (^18^ε) during anammox using a highly enrichment culture of the marine representative *Ca.* Scalindua sp. Previous studies have shown that the *δ*^18^O of NO_3_^−^ (where two of the three O atoms in NO_3_^−^ are derived from water) produced in aerobic nitrification generally converges toward the *δ*^18^O of ambient water due to isotopic equilibrium between NO_2_^−^ and H_2_O and kinetic isotope effects during oxygen uptake from molecular oxygen and H_2_O. The present study further demonstrates that *δ*^18^O_NO3-_ converges near the *δ*^18^O of ambient water as a result of significant O isotope exchange between NO_2_^−^ and H_2_O, even during the anaerobic oxidation of NO_2_^−^ to NO_3_^−^ (where only one O atom is incorporated from H_2_O into NO_3_^−^) in anammox metabolism. Therefore, caution is needed when using *δ*^18^O_NO3-_ and *δ*^18^O_NO2-_ as geochemical markers to evaluate their potential contribution to fixed nitrogen losses in the ocean, as the isotope signals of anammox and denitrification reactions may be altered even under anoxic conditions. Further studies are required to determine the extent to which anammox-mediated oxygen isotope exchange occurs in natural environments and its impact on *δ*^18^O_NO3-_ and *δ*^18^O_NO2-_.

## Supplementary Material

Kobayashi_et_al_Supplementary_Materials_wraf115

## Data Availability

All the data underlying this manuscript are available in the main text and/or the Supplementary Materials. The R code will be available at ZENODO.
