# Supplementary material for: Oxygen isotope fractionation during anaerobic ammonium oxidation by the marine representative Candidatus Scalindua sp
Source: ISME J. 2025 Jun 2;19(1):wraf115. doi: 10.1093/ismejo/wraf115 (PMC12203076; doi:10.1093/ismejo/wraf115)
Supplement: Kobayashi_et_al_Supplementary_Materials_wraf115 [file kobayashi_et_al_supplementary_materials_wraf115.pdf]

Supplementary Materials for

Oxygen isotope fractionation during anaerobic ammonium oxidation by the marine representative *Candidatus* Scalindua sp.

Kanae Kobayashi,[1,2] Kazuya Nishina,[3] Keitaro Fukushima,[4] Yuji Onishi,[4]

Akiko Makabe,[2] Mamoru Oshiki,[1] Keisuke Koba,[4] and Satoshi Okabe[1]*

[1] Division of Environmental Engineering, Faculty of Engineering, Hokkaido University, North-13, West-8, Kita-ku, Sapporo, Hokkaido, 060-8628, Japan.

[2] Institute for Extra-cutting-edge Science and Technology Avant-garde Research (X-star), Japan Agency for Marine-Earth Science and Technology (JAMSTEC), Yokosuka, 237-0061, Japan.

[3] Biogeochemical Cycle Modeling and Analysis Section, Earth System Division, National Institute for Environmental Studies, Onogawa, Tsukuba, 305-8506, Japan

[4] Center for Ecological Research, Kyoto University, Otsu, Shiga, 520-2113, Japan.

*Corresponding author: Satoshi Okabe

Division of Environmental Engineering, Faculty of Engineering, Hokkaido University

North-13, West-8, Kita-ku, Sapporo, Hokkaido 060-8628, JAPAN

Email: sokabe@eng.hokudai.ac.jp

This PDF file includes:

Supplementary Text

Figs. S1 to **S15**

Tables S1 to **S5**

Supplementary Text

Nitrogen isotope effects model
Determine the ^{14}N flux by the change of concentration

Anammox bacteria oxidize NH_4^+ directly to N_2 gas with NO_2^- as the terminal electron acceptor in the absence of oxygen, and NO_2^- is concomitantly oxidized to NO_3^- as shown in the following stoichiometric equation [1].

$$1NH_4^+ + 1.146NO_2^- + 0.071HCO_3^- + 0.057H^+ \rightarrow 0.986N_2 + 0.161NO_3^- + 0.071CH_{1.74}O_{0.31}N_{0.20} + 2.002H_2O \cdots (S1)$$

The main three fluxes of anammox reaction were labeled as follows: NH_4^+ oxidation to N_2 (AMO), NO_2^- reduction to N_2 (NIR) and NO_2^- oxidation to NO_3^- (NXR). The amount of ^{14}N (99.6337%) is much larger than that of ^{15}N (0.36630%). Thus, the ^{14}N flux was represented by the change of concentration in this model. In brief, the simulated $^{14}NNH_4^+$ pool is influenced by NH_4^+ oxidation (^{14}NAMO). The NO_2^- pool reflects the balance of consumption by NO_2^- reduction (^{14}NNIR), as well as NO_2^- oxidation (^{14}NNXR). The NO_3^- pool is influenced by NO_2^- oxidation (^{14}NNXR).

$$^{14}\text{NAMO}([\text{N}]/\text{time}) = {}^{14}\text{N flux of } NH_4^+ \text{ oxidation to } N_2 \cdots (S2)$$

$$^{14}\text{NNIR}([\text{N}]/\text{time}) = {}^{14}\text{N flux of } NO_2^- \text{ reduction to } N_2 \cdots (S3)$$

$$^{14}\text{NNXR}([\text{N}]/\text{time}) = {}^{14}\text{N flux of } NO_2^- \text{ oxidation to } NO_3^- \cdots (S4)$$

The consumption rate of NH_4^+ and NO_2^- and production rate of NO_3^- were determined by the slope of regression line between time (min) vs. concentration (mmol-N/L) plots (**Figs. 2 A** to **D**, and **Figs. S1** to **S4**, **A** to **C**).

$$(NH_4^+)_{\text{cons.}} = -\text{slope}\,[NH_4^+] \cdots (S5)$$

$$(NO_2^-)_{\text{cons.}} = -\text{slope}[NO_2^-] \cdots (S6)$$

$$(NO_3^-)_{\text{prod.}} = \text{slope}\,[NO_3^-] \cdots (S7)$$

The flux of NH_4^+ oxidation (^{14}NAMO) is designated as follows.

$$^{14}\text{NAMO} = (NH_4^+)_{\text{cons.}} \cdots (S8)$$

The flux of NO_2^- reduction to N_2 (^{14}NNIR) is designated as follows.

$$^{14}\text{NNIR} = (NO_2^-)_{\text{cons.}} - (NO_3^-)_{\text{cons.}} \cdots (S9)$$

The flux of NO_2^- oxidation to NO_3^- (^{14}NNXR) is designated as follows.

$$^{14}\text{NNXR} = (\text{NO}_3^-)_{\text{prod.}} \cdots (\text{S10})$$

Each ^{14}N flux can be determined by taking mass balance and fitted to the actual measured time course changes in NH_4^+, NO_2^-, and NO_3^- concentrations.

$$\frac{\text{d}}{\text{dt}}\left[{}^{14}\text{N}_{\text{NH}_4^+}\right] = -{}^{14}\text{NAMO} \cdots (\text{S11})$$

$$\frac{\text{d}}{\text{dt}}\left[{}^{14}\text{N}_{\text{NO}_2^-}\right] = -{}^{14}\text{NNIR} - {}^{14}\text{NNXR} \cdots (\text{S12})$$

$$\frac{\text{d}}{\text{dt}}\left[{}^{14}\text{N}_{\text{NO}_3^-}\right] = +{}^{14}\text{NNXR} \cdots (\text{S13})$$

Calculation of ^{15}N flux based on nitrogen isotope composition

The definition of nitrogen isotope composition is as follows:

$$\delta^{15}\text{N} = \left(\frac{^{15}R_{\text{sample}}}{^{15}R_{\text{air}}} - 1\right) \times 1000\ (‰) \cdots (\text{S14})$$

Arrange Eq. S14

$$^{15}\text{R}_{\text{sample}} = \left(\frac{\delta^{15}\text{N}}{1000} + 1\right) \times {}^{15}\text{R}_{\text{air}} \cdots (\text{S15})$$

^{15}R is a ratio of heavy isotope and light isotope:

$$^{15}\text{R} = \frac{\left[{}^{15}\text{N}\right]}{\left[{}^{14}\text{N}\right]} \cdots (\text{S16})$$

$^{15}R_{air}$ is a ^{15}R value of international N reference standards ($^{15}R_{air}$ = 0.0036765).

^{15}R of each nitrogen compound is defined as follows:

$$^{15}\text{R}_{\text{NH}_4^+} = \frac{\left[{}^{15}\text{N}_{\text{NH}_4^+}\right]}{\left[{}^{14}\text{N}_{\text{NH}_4^+}\right]} \cdots (\text{S17})$$

$$^{15}\text{R}_{\text{NO}_2^-} = \frac{\left[{}^{15}\text{N}_{\text{NO}_2^-}\right]}{\left[{}^{14}\text{N}_{\text{NO}_2^-}\right]} \cdots (\text{S18})$$

$$^{15}\text{R}_{\text{NO}_3^-} = \frac{\left[{}^{15}\text{N}_{\text{NO}_3^-}\right]}{\left[{}^{14}\text{N}_{\text{NO}_3^-}\right]} \cdots (\text{S19})$$

The definition of isotope effect (ε) is as follows:

$$\varepsilon = (\alpha - 1) \times 1000\ (‰) \cdots (S20)$$

α is an isotope fractionation factor explained by following equation.

$$^{15}\alpha = \frac{\frac{^{14}\mathrm{N\ flux}}{[^{14}\mathrm{N}]}}{\frac{^{15}\mathrm{N\ flux}}{[^{15}\mathrm{N}]}} \cdots (S21)$$

Arrange the Eq. S21.

$$^{15}\mathrm{N\ flux} = \frac{[^{15}\mathrm{N}]}{[^{14}\mathrm{N}]} \times \frac{^{14}\mathrm{Nflux}}{^{15}\alpha} \cdots (S22)$$

From the Eq. S16

$$^{15}\mathrm{Nflux} = {}^{15}\mathrm{R} \times \frac{^{14}\mathrm{Nflux}}{^{15}\alpha} \cdots (S23)$$

As a result, the ^{15}N flux is explained by ^{15}R, ^{14}N flux and isotope fractionation factor ($^{15}\alpha$). ^{15}N flux by each anammox reaction is defined as following.

$$^{15}\mathrm{NAMO} = {}^{15}\mathrm{R}_{\mathrm{NH_4^+}} \times \frac{^{14}\mathrm{NAMO}}{^{15}\alpha_{AMO}} \cdots (S24)$$

$$^{15}\mathrm{NNIR} = {}^{15}\mathrm{R}_{\mathrm{NO_2^-}} \times \frac{^{14}\mathrm{NNIR}}{^{15}\alpha_{NIR}} \cdots (S25)$$

$$^{15}\mathrm{NNXR} = {}^{15}\mathrm{R}_{\mathrm{NO_2^-}} \times \frac{^{14}\mathrm{NNXR}}{^{15}\alpha_{NXR}} \cdots (S26)$$

Those three parameters ($^{15}\alpha_{AMO}$, $^{15}\alpha_{NIR}$, and $^{15}\alpha_{NXR}$) were determined by taking mass balance of ^{15}N of N compounds and modeled to fit to the actual measured time course changes in ^{15}N of NH_4^+, NO_2^-, and NO_3^-.

$$\frac{d}{dt}[^{15}\mathrm{N}_{\mathrm{NH_4^+}}] = -{}^{15}\mathrm{NAMO} = -{}^{15}\mathrm{R}_{\mathrm{NH_4^+}} \times \frac{^{14}\mathrm{NAMO}}{^{15}\alpha_{AMO}} \cdots (S27)$$

$$\frac{d}{dt}[^{15}\mathrm{N}_{\mathrm{NO_2^-}}] = -{}^{15}\mathrm{NNIR} - {}^{15}\mathrm{NNXR} = -{}^{15}\mathrm{R}_{\mathrm{NO_2^-}} \times \frac{^{14}\mathrm{NNIR}}{^{15}\alpha_{NIR}} - {}^{15}\mathrm{R}_{\mathrm{NO_2^-}} \times \frac{^{14}\mathrm{NNXR}}{^{15}\alpha_{NXR}} \cdots (S28)$$

$$\frac{d}{dt}[^{15}\mathrm{N}_{\mathrm{NO_3^-}}] = +{}^{15}\mathrm{NNXR} = +{}^{15}\mathrm{R}_{\mathrm{NO_2^-}} \times \frac{^{14}\mathrm{NNXR}}{^{15}\alpha_{NXR}} \cdots (S29)$$

In order to calculate $^{15}\alpha_{AMO}$, $^{15}\alpha_{NIR}$, and $^{15}\alpha_{NXR}$, the differential equations were fitted using the R package FME [2]. The R package FME is a modeling package designed to confront a mathematical model with data. It includes algorithms for sensitivity and Monte Carlo analysis, parameter identifiability, and model fitting and provides a Markov-chain based method to estimate parameter confidence intervals. All simulations were run using this package.

Oxygen isotope effect model

Determination of ^{16}O flux based on the change of concentration

The amount of ^{16}O (99.76206%) of nitrogen compounds is much larger than that of ^{18}O (0.0020004%). Thus, the ^{16}O flux was represented by the change in concentration in this model. In brief, the NO_2^- pool reflects the balance of consumption by NO_2^- reduction (NIR), as well as NO_2^- oxidation (NXR). The NO_3^- pool is influenced by NXR. During NO_2^- oxidation, one oxygen atom is incorporated from H_2O. The oxygen incorporation flux is defined as ^{16}ONXR, H_2O.

$$^{16}\mathrm{ONIR}([\mathrm{O}]/\mathrm{time}) = \ ^{16}\mathrm{O\ flux\ of\ NO_2^-\ reduction\ to\ N_2} \cdots (S30)$$

$$^{16}\mathrm{ONXR}([\mathrm{O}]/\mathrm{time}) = \ ^{16}\mathrm{O\ flux\ of\ NO_2^-\ oxidation\ to\ NO_3^-} \cdots (S31)$$

$$^{16}\mathrm{ONXR, H_2O}\ ([\mathrm{O}]/\mathrm{time}) = \begin{matrix} ^{16}\mathrm{O\ flux\ of\ oxygen\ incorporation} \\ \mathrm{from\ H_2O\ during\ NO_2^-\ oxidation} \end{matrix} \cdots (S32)$$

^{16}O flux could be defined based on ^{14}N flux.

When one NO_2^- is reduced to N_2, two oxygen molecules are lost from NO_2^- pool.

$$^{16}\mathrm{ONIR} = \ ^{14}\mathrm{NNIR} \times 2 \cdots (S33)$$

When one NO_2^- is oxidized to NO_3^-, two oxygen molecules are lost from NO_2^- pool.

$$^{16}\mathrm{ONXR} = \ ^{14}\mathrm{NNXR} \times 2 \cdots (S34)$$

When one NO_2^- is oxidized to NO_3^-, one oxygen molecule is incorporated from H_2O.

$$^{16}\mathrm{ONXR, H_2O} = \frac{^{16}\mathrm{ONXR}}{2} \cdots (S35)$$

^{16}O mass balance was taken by following equations.

$$\frac{\mathrm{d}}{\mathrm{dt}}\left[^{16}\mathrm{O}_{\mathrm{NO_2^-}}\right] = -^{16}\mathrm{ONIR} - \ ^{16}\mathrm{ONXR} \cdots (S36)$$

$$\frac{\mathrm{d}}{\mathrm{dt}}\left[^{16}\mathrm{O}_{\mathrm{NO_3^-}}\right] = +^{16}\mathrm{ONXR} + \ ^{16}\mathrm{ONXR, H_2O} \cdots (S37)$$

Calculation of ^{18}O flux by anammox based on oxygen isotope composition

The definition of oxygen isotope composition is as follows:

$$\delta^{18}\mathrm{O} = \left(\frac{^{18}\mathrm{R}_{\mathrm{sample}}}{^{18}\mathrm{R}_{\mathrm{VSMOW}}} - 1\right) \times 1000\ (‰) \cdots (S38)$$

Arrange the Eq. S38.

$$^{18}\mathrm{R}_{\mathrm{sample}} = \left(\frac{\delta^{18}\mathrm{O}}{1000} + 1\right) \times {}^{18}\mathrm{R}_{\mathrm{VSMOW}} \cdots (S39)$$

Where $^{18}R = {}^{18}O/{}^{16}O$ and VSMOW refers to Vienna Standard Mean Ocean Water
$^{18}R_{VSMOW} = 0.00200517$
^{18}R of each nitrogen compounds is defined as follows:

$$^{18}\mathrm{R}_{\mathrm{NO_2^-}} = \frac{[^{18}\mathrm{O}_{\mathrm{NO_2^-}}]}{[^{16}\mathrm{O}_{\mathrm{NO_2^-}}]} \cdots (S40)$$

$$^{18}\mathrm{R}_{\mathrm{NO_3^-}} = \frac{[^{18}\mathrm{O}_{\mathrm{NO_3^-}}]}{[^{16}\mathrm{O}_{\mathrm{NO_3^-}}]} \cdots (S41)$$

$$^{18}\mathrm{R}_{\mathrm{H_2O}} = \left(\frac{\delta^{18}\mathrm{O}_{\mathrm{H_2O}}}{1000} + 1\right) \times {}^{18}\mathrm{R}_{\mathrm{VSMOW}} \cdots (S42)$$

The ^{18}O flux is explained by ^{18}R, ^{16}O flux and isotope fractionation factor ($^{18}\alpha$). ^{18}O flux from each anammox reaction, as well as the ^{15}N flux, is defined as follows

$$^{18}\mathrm{ONIR} = {}^{18}\mathrm{R}_{\mathrm{NO_2^-}} \times \frac{^{16}\mathrm{ONIR}}{^{18}\alpha_{\mathrm{NIR}}} \cdots (S43)$$

$$^{18}\mathrm{ONXR} = {}^{18}\mathrm{R}_{\mathrm{NO_2^-}} \times \frac{^{16}\mathrm{ONXR}}{^{18}\alpha_{\mathrm{NXR}}} \cdots (S44)$$

The ^{18}O flux due to oxygen incorporation from water during NO_2^- oxidation to NO_3^- ($^{18}ONXR, H_2O$) could be described as follows [3].

$$^{18}\mathrm{ONXR,H_2O} = \frac{^{18}\mathrm{R}_{\mathrm{H_2O}} \times \frac{^{16}\mathrm{ONXR}}{2}}{^{18}\alpha_{\mathrm{NXR,H_2O}}} \cdots (S45)$$

Abiotic oxygen isotope exchange between nitrite and water

The oxygen atom of NO_2^- is abiotically exchanged with H_2O [4]. The $\delta^{18}O$ of newly produced NO_3^- is also known to be sensitive to $\delta^{18}O$ of ambient water and the equilibrium isotope effects between NO_2^- and H_2O [3]. However, the experimentally determined NO_2^- equilibration rates (k_{eq}) [5, 6] and equilibrium isotope effects ($^{18}\varepsilon_{eq}$) [4, 5, 6] under relevant environmental conditions remain quite limited. In our previous work, we determined the rate of abiotic O isotope exchange between NO_2^- and H_2O ($k_{eq, abio}$) and equilibrium isotope effects ($^{18}\varepsilon_{eq, abio}$) under growth condition of "*Ca.* Scalindua sp." at variety of $\delta^{18}O_{H2O}$ values conditions ($\delta^{18}O_{H2O}$ = -12.6, 25.9, 56.7, and 110.1‰) in a series of laboratory experiments [7]. The abiotic oxygen isotope exchange between NO_2^- and H_2O was described by using previously determined k_{eq} and $^{18}\varepsilon_{eq}$.

The approach of $\delta^{18}O_{NO2}$ to isotope equilibrium ($\delta^{18}O_{NO2-, eq}$) is modeled as a following exponential form [5], where $\delta^{18}O_{NO2-, initial}$ and $\delta^{18}O_{NO2-, eq}$ are the value of $\delta^{18}O_{NO2-}$ at initial and equilibrium state, respectively:

$$\delta^{18}\mathrm{O}_{\mathrm{NO_2^-}}(\mathrm{t}) = \delta^{18}\mathrm{O}_{\mathrm{NO_2^-,eq}} + \left(\delta^{18}\mathrm{O}_{\mathrm{NO_2^-,initial}} - \delta^{18}\mathrm{O}_{\mathrm{NO_2^-,eq}}\right) \times \exp(-k\mathrm{t}) \cdots (S46)$$

According to equation (S46), the value of $\delta^{18}O_{NO2-}$ (t) is determined by time, $\delta^{18}O_{NO2-, initial}$ and $\delta^{18}O_{NO2-, eq}$. In order to determine the change in $\delta^{18}O_{NO2-}$ per unit time, the equation (S46) was differentiated by time.

$$\frac{\mathrm{d}}{\mathrm{dt}}\left(\delta^{18}\mathrm{O}_{\mathrm{NO_2^-}}(t)\right) = -k \times (\delta^{18}\mathrm{O}_{\mathrm{NO_2^-,initial}} - \delta^{18}\mathrm{O}_{\mathrm{NO_2^-,eq}}) \times \exp(-k\mathrm{t}) \cdots (S47)$$

According to equation (S47), the change in $\delta^{18}O_{NO2-}$ is determined by difference between $\delta^{18}O_{NO2-, initial}$ and $\delta^{18}O_{NO2-, eq}$. We defined $\delta^{18}O_{NO2-}$ (T) is the value of $\delta^{18}O_{NO2-}$ at time T in the batch incubation.

$$\delta^{18}O_{NO_2^-}(T) = \left(\frac{\frac{^{18}O_{NO_2^-}}{^{16}O_{NO_2^-}}}{^{18}R_{VSMOW}} - 1\right) \times 1000\ (‰) \cdots (S48)$$

The change in $\delta^{18}O_{NO2-}$ per unit time ($\delta^{18}O_{NO2-}(DT)_{exchange}$) at time T could be given by the following equation, where the $\delta^{18}O_{NO2-, eq}$ is the value of $\delta^{18}O_{NO2-}$ at the equilibrium state.

$$\delta^{18}\mathrm{O}_{\mathrm{NO_2^-}}(\mathrm{DT})_{\mathrm{exchange}} =- k \times \left(\delta^{18}\mathrm{O}_{\mathrm{NO_2^-}}(\mathrm{T}) - \delta^{18}\mathrm{O}_{\mathrm{NO_2^-,eq}}\right) \times \exp(-k \times \mathrm{DT}) \cdots (S49)$$

Time = 1 min = 1DT

In our numerical model, ^{18}O mass balance (d/dt[$^{18}O_{NO2-}$]) was taken. Therefore, equation (S49) was rearranged to the change in ^{18}O pool of NO_2^-.

Example

$$\delta^{18}O = \left(\frac{^{18}R_{sample}}{^{18}R_{VSMOW}} - 1\right) \times 1000\ (‰) \cdots (S50)$$

$$^{18}R_{sample} = \left(\frac{\delta^{18}O}{1000} + 1\right) \times {}^{18}R_{VSMOW} \cdots (S51)$$

Where, $^{18}R = {}^{18}O / {}^{16}O$ (mole ratio)

$$^{18}O_{sample} = \left(\frac{\delta^{18}O}{1000} + 1\right) \times {}^{18}R_{VSMOW} \times {}^{16}O_{sample} \cdots (S52)$$

As same as example, the change in ^{18}O pool of NO_2^- ($^{18}O_{NO2-}(DT)_{exchange}$) could be presented by following equation.

$$^{18}O_{NO_2^-}(DT)_{exchange} = \left(\frac{\delta^{18}O_{NO_2^-}(DT)_{exchange}}{1000} + 1\right) \times {}^{18}R_{VSMOW} \times {}^{16}O_{NO_2^-} \cdots (S53)$$

When $\delta^{18}O_{NO2-}$ reaches the equilibrium state, the value of $^{18}O_{NO2-}(DT)_{exchange}$ should be zero. At the equilibrium state, $\delta^{18}O_{NO2-}(DT)_{exchange}$ is zero. Therefore, this equation (S53) was corrected as follows.

$$^{18}O_{NO_2^-}(DT)_{exchange} = \left(\frac{\delta^{18}O_{NO_2^-}(DT)_{exchange}}{1000} + 1\right) \times {}^{18}R_{VSMOW} \times {}^{16}O_{NO_2^-} - \left(\frac{0}{1000} + 1\right) \times {}^{18}R_{VSMOW} \times {}^{16}O_{NO_2^-} \cdots (S54)$$

However, during anammox reaction, not only abiotic oxygen isotope exchange but also reactions of NO_2^- oxidation and NO_2^- reduction affect $\delta^{18}O_{NO2-}$. As a result, the behavior of $\delta^{18}O_{NO2-}$ is different from abiotic oxygen isotope exchange between NO_2^- and H_2O.

Microbially catalyzed oxygen isotope exchanges between NO_2^- and H_2O

From the observation of changes in $\delta^{18}O_{NO2-}$ in batch incubation experiments (**Fig. 4** and **Fig. S8**), anammox bacteria accelerated oxygen isotope exchange between NO_2^- and H_2O. Therefore, we defined the reaction rate constant of anammox-mediated oxygen isotope exchange between NO_2^- and H_2O as $k_{eq,\ AMX}$ for the following model simulation (**Fig. 1**). The value of equilibrium isotope effect during anammox reaction ($^{18}\varepsilon_{eq,\ AMX}$) was assumed to be the same value as abiotic equilibrium isotope effect ($^{18}\varepsilon_{eq,\ abio}$ = 11.9‰) [7] (**Fig. 1**). As same as abiotic oxygen isotope exchange (Eq. S49), the change in $\delta^{18}O_{NO2-}$ per unit time during anammox reaction ($^{18}O_{NO2-}(DT)_{exchange,\ AMX}$) is given by following equation.

$$\delta^{18}O_{NO_2^-}(DT)_{exchange,AMX} = -k_{eq,AMX} \times \left(\delta^{18}O_{NO_2^-}(T) - \delta^{18}O_{NO_2^-,eq}\right) \times \exp\left(-k_{eq,AMX} \times DT\right) \cdots (S55)$$

The change in ^{18}O pool of NO_2^- ($^{18}O_{NO2-}(DT)_{exchange, AMX}$) could be presented by following equation.

$$^{18}O_{NO_2^-}(DT)_{exchange,AMX} = \left(\frac{\delta^{18}O_{NO_2^-}(DT)_{exchange,AMX}}{1000}+1\right) \times {}^{18}R_{VSMOW} \times {}^{16}O_{NO_2^-} - \left(\frac{0}{1000}+1\right) \times {}^{18}R_{VSMOW} \times {}^{16}O_{NO_2^-} \cdots (S56)$$

During anammox reaction, $\delta^{18}O_{NO2-}$ is influenced by NO_2^- reduction, NO_2^- oxidation, abiotic oxygen isotope exchange, and anammox-mediated oxygen isotope exchange. $\delta^{18}O_{NO3-}$ is influenced by O atom incorporation from H_2O during NO_2^- oxidation and NO_2^- oxidation. Thus, the ^{18}O pools of NO_2^- and NO_3^- could be shown as follows.

$$\frac{d}{dt}\left[{}^{18}O_{NO_2^-}\right] = -{}^{18}ONIR - {}^{18}ONXR + 1 \times {}^{18}O_{NO_2^-}(DT)_{exchange} + 1 \times {}^{18}O_{NO_2^-}(DT)_{exchange,AMX} \cdots (S57)$$

$$\frac{d}{dt}\left[{}^{18}O_{NO_3^-}\right] = {}^{18}NXR, H_2O + {}^{18}ONXR \cdots (S58)$$

Calculation of the fraction of nitrite oxygen atoms exchanged with water during anammox-mediated nitrite oxidation

The $\delta^{18}O_{NO3-}$ produced when nitrite oxidation goes to completion ($\delta^{18}O_{NO3-,final}$) can be defined as follows [5, 8]:

$$\delta^{18}O_{NO3-,final} = \frac{2}{3}\left[(1-x)\delta^{18}O_{NO2-,initial} + x\left(\delta^{18}O_{H2O} + {}^{18}\varepsilon_{eq}\right)\right] + \frac{1}{3}\left(\delta^{18}O_{H2O} - {}^{18}\varepsilon_{H2O}\right)$$

where x is the fraction of nitrite oxygen atoms that have equilibrated with water during NO_2^- oxidation, $\delta^{18}O_{NO2-,initial}$ is the $\delta^{18}O$ of nitrite at the start of the experiment, $^{18}\varepsilon_{eq}$ is the equilibrium isotope effect between NO_2^- and H_2O, and $^{18}\varepsilon_{H2O}$ is the isotope effect associated with the incorporation of an oxygen atom from H_2O during NO_2^- oxidation.

If this equation is arranged to group $\delta^{18}O_{H2O}$ terms, it can be used to interpret the slope and intercept of the linear regression of $\delta^{18}O_{NO3-,final}$ vs. $\delta^{18}O_{H2O}$:

$$\delta^{18}O_{NO3-,final} = \left[\frac{2}{3}x + \frac{1}{3}\right]\delta^{18}O_{H2O} + \left\{\frac{2}{3}\left[(1-x)\delta^{18}O_{NO2-,initial} + {}^{18}\varepsilon_{eq} \cdot x\right] - \frac{1}{3}\,{}^{18}\varepsilon_{H2O}\right\}$$

Thus, for a plot of $\delta^{18}O_{NO3-,final}$ vs. $\delta^{18}O_{H2O}$ (**Fig. 5**), the slope should be equal to $\left[\frac{2}{3}x + \frac{1}{3}\right]$. If there is no exchange (x=0), a slope of 0.33 would be expected, and if there is full exchange (x=1) the slope would be 1. In **Figure 5**, the slope is 0.56, then

$$\left[\frac{2}{3}x + \frac{1}{3}\right] = 0.56$$

$$x = \frac{3 \cdot 0.56 - 1}{2} = 0.34$$

This indicates that 34% of the oxygen atoms in the newly formed NO_3^- were derived from H_2O during the oxidation of NO_2^- to NO_3^-.

Influence of $\delta^{18}O_{H2O}$ on NO_3^- $\Delta\delta^{18}O$: $\Delta\delta^{15}N$ trajectory of during anammox reaction (Model simulation exercise)

The model simulation exercises were conducted to understand the influence of anammox on the behavior of $\Delta\delta^{18}O_{NO3-}$: $\Delta\delta^{15}N_{NO3-}$ using the obtained N and O isotope effects of anammox. In addition, the influence of $\delta^{18}O_{H2O}$ on anammox $\Delta\delta^{18}O_{NO3-}$: $\Delta\delta^{15}N_{NO3-}$ trajectories was evaluated, because the dependence of $\delta^{18}O_{NO2-}$ and $\delta^{18}O_{NO3-}$ on $\delta^{18}O_{H2O}$ was observed.

For model simulation exercises, the $\delta^{18}O_{H2O}$ was set within the range commonly detected in the ocean ($\delta^{18}O_{H2O}$ = -7.7 to 1.8‰) [9]. Due to the variability of $^{18}\varepsilon$, we used the $^{18}\varepsilon$ values obtained from the batch experiment with $\delta^{18}O_{H2O}$ = -12.6‰, which is closest to the set range of $\delta^{18}O_{H2O}$. We also set the initial $\delta^{15}N_{NO3-}$ to 5‰ and $\delta^{18}O_{NO3-}$ to 2‰, which are close to values commonly observed in the deep ocean [10]. Since $\delta^{15}N_{NO2-}$ was detected in a wide range and known to be 5 to 100‰ lower than $\delta^{15}N_{NO3-}$ [10], we set the initial $\delta^{15}N_{NO2-}$ to 0‰. Assuming that isotopic equilibrium has been reached between NO_2^- and H_2O, the initial value of $\delta^{18}O_{NO2-}$ was set to 8.95‰ by adding the equilibrium isotopic fraction of 11.9‰ to the median $\delta^{18}O_{H2O}$ value of -2.95‰ ((-7.7 + 1.8) / 2). All the parameters used for the model simulation exercises are listed in **Table S5**.

Reference

1. Lotti T et al. Physiological and kinetic characterization of a suspended cell anammox culture. *Water Res* 2014;**60**:1–14. 10.1016/j.watres.2014.04.017
2. Soetaert K, Petzoldt T. Inverse modelling, sensitivity and monte carlo analysis in R using package FME. *J Stat Softw* 2010;**33**:1–28. 10.18637/jss.v033.i03
3. Wunderlich A, Meckenstock RU, Einsiedl F. A mixture of nitrite-oxidizing and denitrifying microorganisms affects the $\delta^{18}O$ of dissolved nitrate during anaerobic microbial denitrification depending on the $\delta^{18}O$ of ambient water. *Geochim Cosmochim Acta* 2013;**119**:31–45. 10.1016/j.gca.2013.05.028
4. Li S et al. Denitrification fractionates N and O isotopes of nitrate following a ratio independent of carbon sources in freshwaters. *Environ Microbiol* 2023;**25**:2404-15.10.1111/1462-2920.16468
5. Buchwald C, Casciotti K. Isotopic ratios of nitrite as tracers of the sources and age of oceanic nitrite. *Nat Geosci* 2013;**6**:1–6. 10.1038/ngeo1745
6. Nishizawa M et al. Nitrogen and oxygen isotope effects of ammonia oxidation by thermophilic *Thaumarchaeota* from a geothermal water stream. *Appl Environ Microbiol* 2016;**82**:4492–504. 10.1128/AEM.00250-16
7. Kobayashi K et al. Influence of $\delta^{18}O$ of water on measurements of $\delta^{18}O$ of nitrite and nitrate. *Rapid*

Commun Mass Spectrom 2020;**35**:e8979. 10.1002/rcm.8979
8. Casciotti KL, McIlvin M, Buchwald C. Oxygen isotopic exchange and fractionation during bacterial ammonia oxidation. *Limnol Oceanogr* 2010;**55**:753–62. 10.4319/lo.2009.55.2.0753
9. Bowen GJ. Isoscapes: Spatial pattern in isotopic biogeochemistry. *Annu Rev Earth Planet Sci* 2010;**38**:161–87. 10.1146/annurev-earth-040809-152429
10. Sigman DM, Fripiat F. Nitrogen isotopes in the ocean. *Encyclopedia of Ocean Sciences* 2019;263–78. 10.1016/B978-0-12-409548-9.11605-7

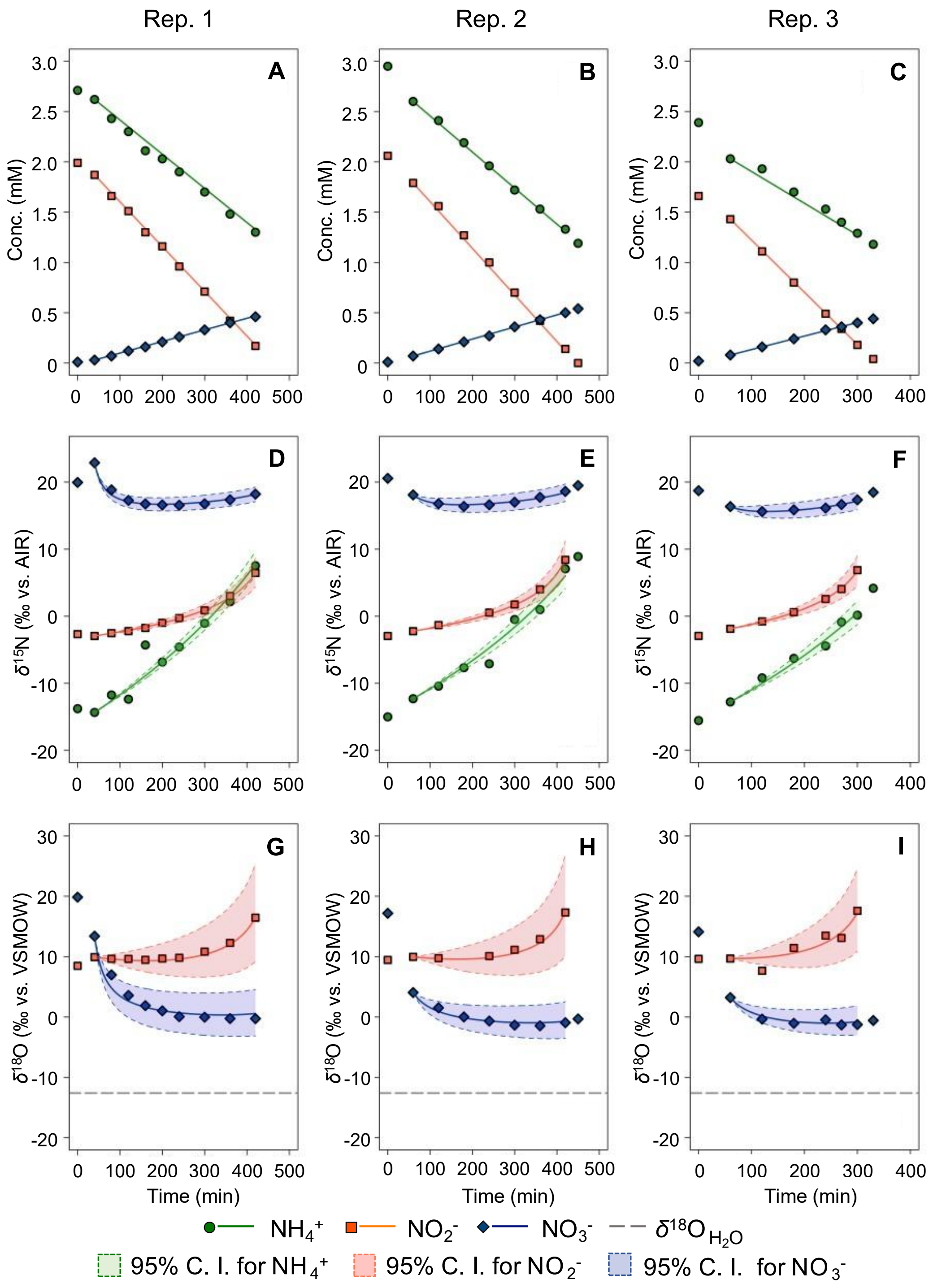

Fig. S1. Changes in concentrations and nitrogen and oxygen isotopic ratios of N compounds over time in batch experiments with $\delta^{18}O_{H2O}$= -12.6‰. Lines represent the changes in the concentrations (**A** - **C**), N isotope signatures (**D** - **F**), and O isotope signatures (**G** - **H**) estimated by a newly constructed MCMC. 95% C.I. indicates 95% credible interval for estimated $\delta^{15}N$ of NH_4^+, NO_2^- and NO_3^- (**D** - **F**) and $\delta^{18}O$ of NO_2^- and NO_3^- (**G** - **H**).

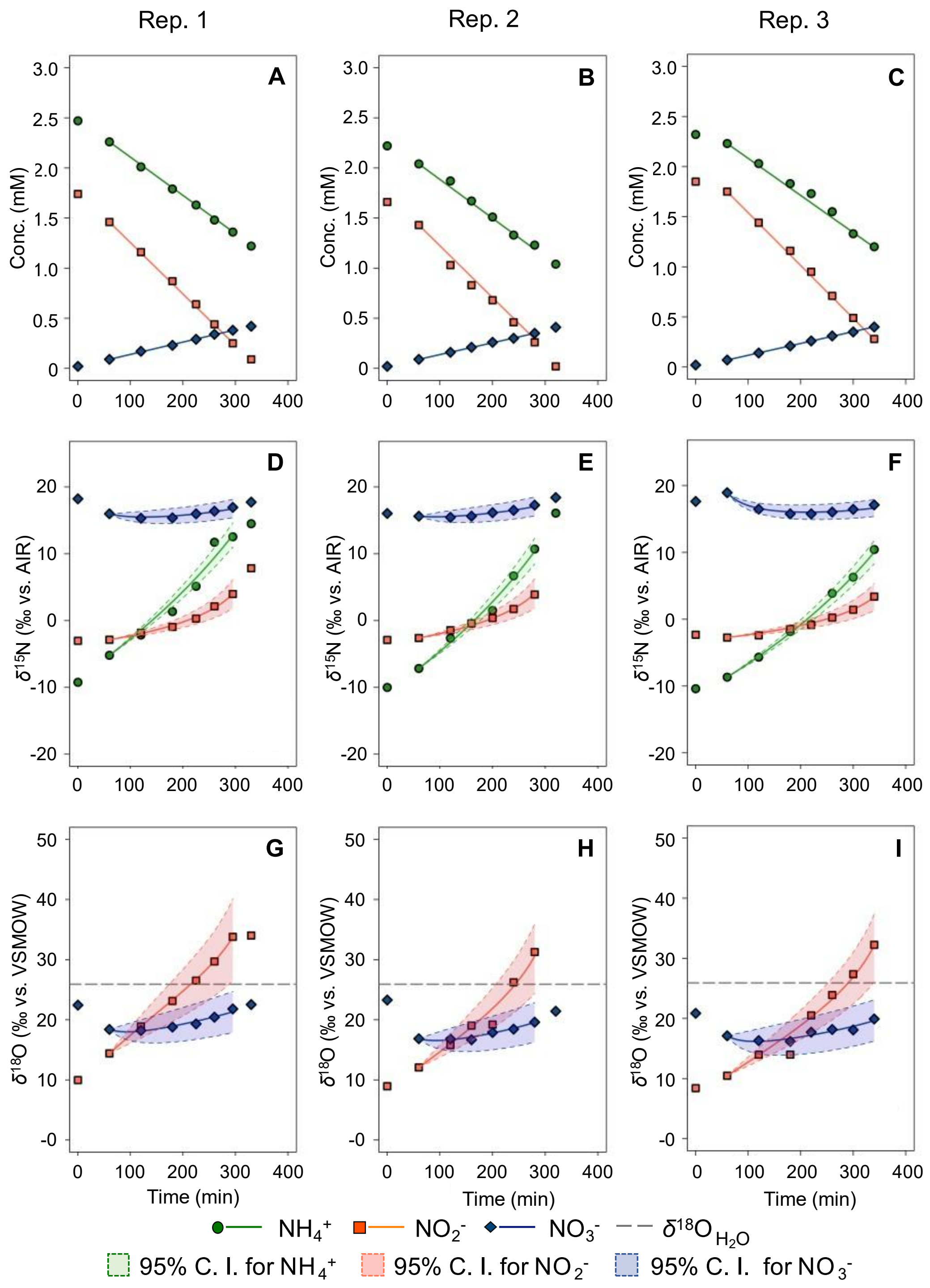

Fig. S2. Changes in concentrations and nitrogen and oxygen isotopic ratios of N compounds over time in batch experiments with $\delta^{18}O_{H2O}$= 25.9‰. Lines represent the changes in the concentrations (**A - C**), N isotope signatures (**D - F**), and O isotope signatures (**G - H**) estimated by a newly constructed MCMC. 95% C.I. indicates 95% credible interval for estimated $\delta^{15}N$ of NH_4^+, NO_2^- and NO_3^- (**D - F**) and $\delta^{18}O$ of NO_2^- and NO_3^- (**G - H**).

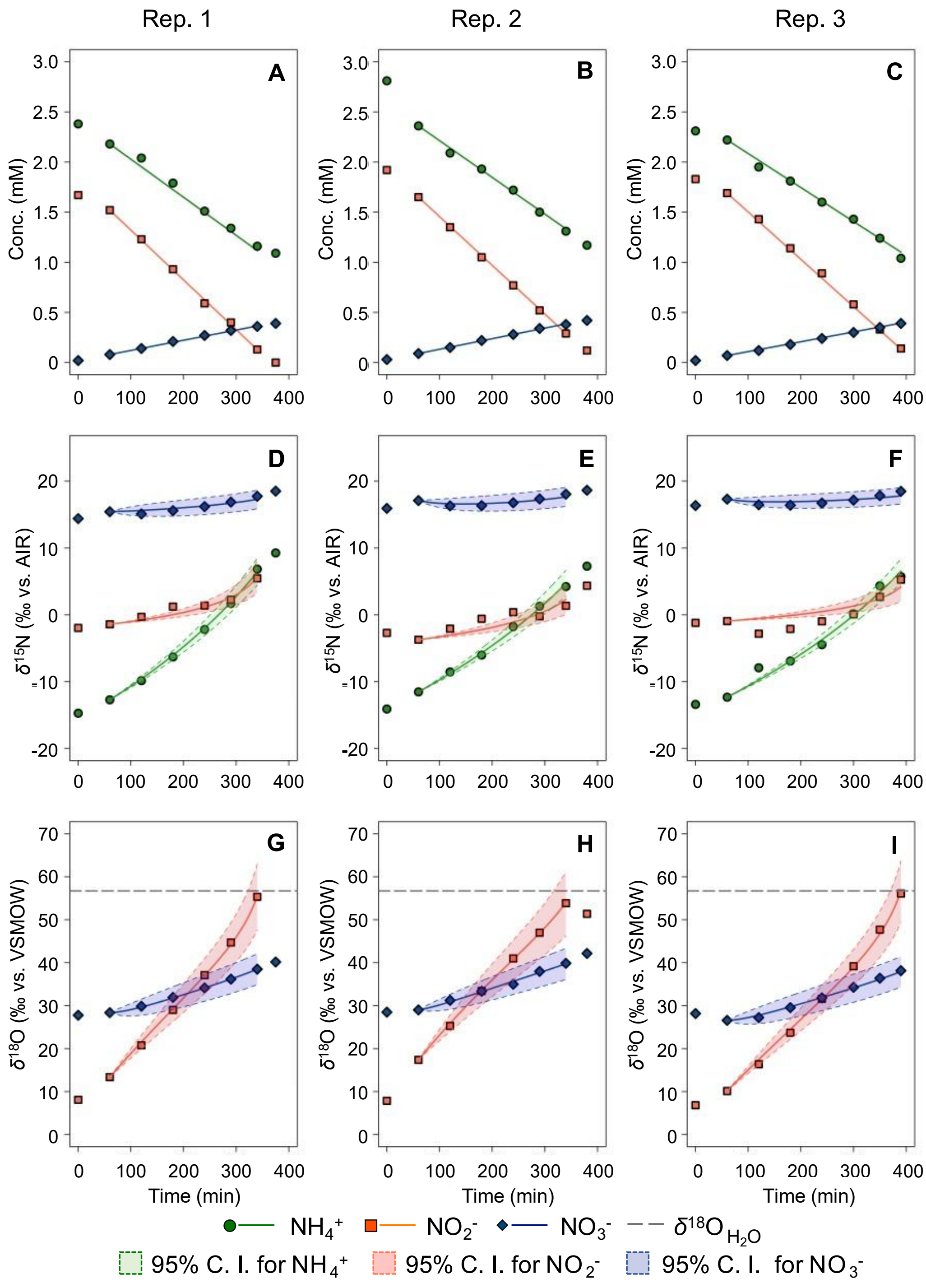

Fig. S3. Changes in concentrations andnitrogen and oxygen isotopic ratios of N compounds over time in batch experiments with $\delta^{18}O_{H2O}$= 56.7‰. Lines represent the changes in the concentrations (**A** - **C**), N isotope signatures (**D** - **F**), and O isotope signatures (**G** - **H**) estimated by a newly constructed MCMC. 95% C.I. indicates 95% credible interval for estimated $\delta^{15}N$ of NH_4^+, NO_2^- and NO_3^- (**D** - **F**) and $\delta^{18}O$ of NO_2^- and NO_3^- (**G** - **H**).

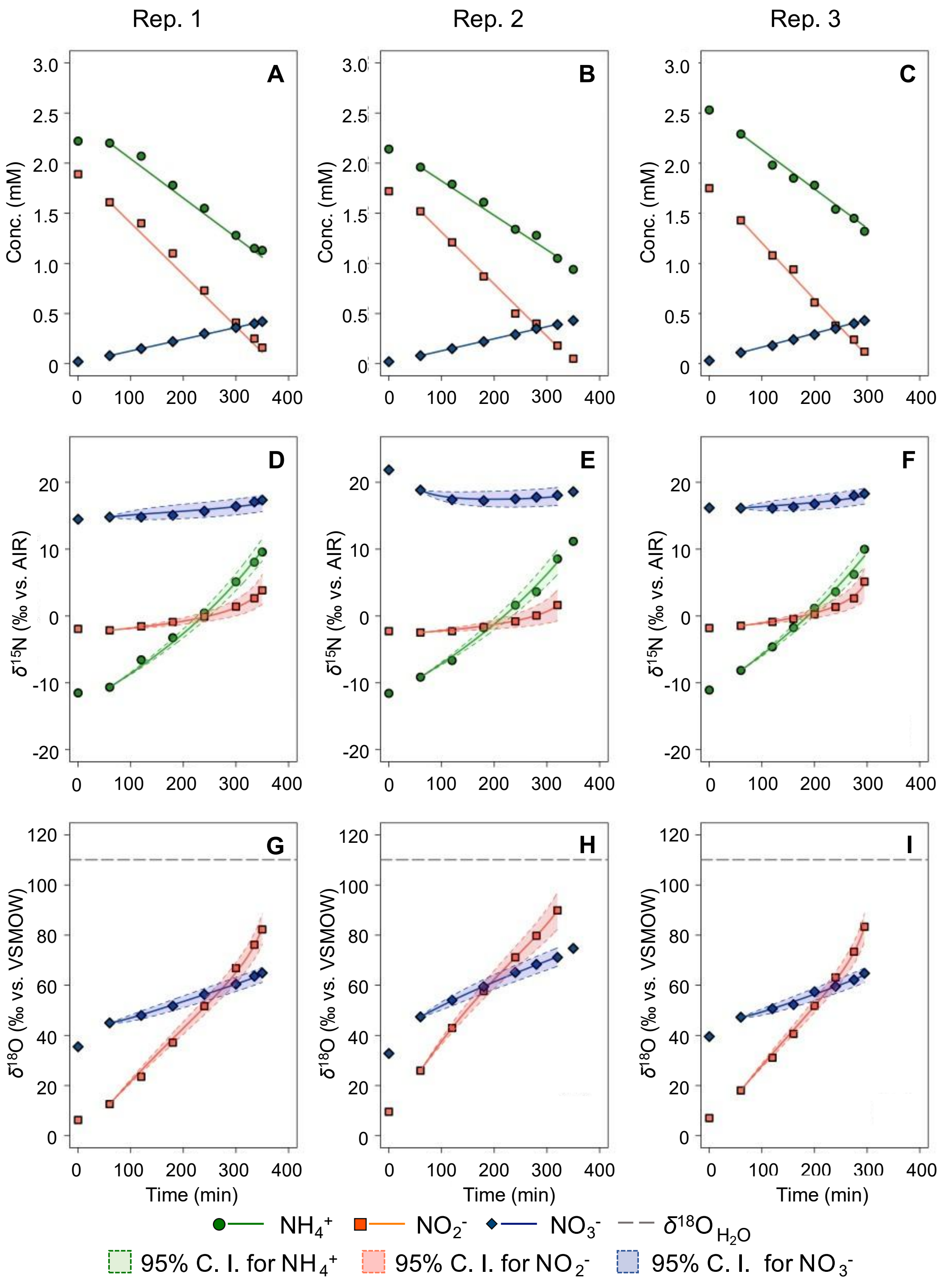

Fig. S4. Changes in concentrations and nitrogen and oxygen isotopic ratios of N compounds over time in batch experiments with $\delta^{18}O_{H2O}$= 110.1‰. Lines represent the changes in the concentrations (**A** - **C**), N isotope signatures (**D** - **F**), and O isotope signatures (**G** - **H**) estimated by a newly constructed MCMC. 95% C.I. indicates 95% credible interval for estimated $\delta^{15}N$ of NH_4^+, NO_2^- and NO_3^- (**D** - **F**) and $\delta^{18}O$ of NO_2^- and NO_3^- (**G** - **H**).

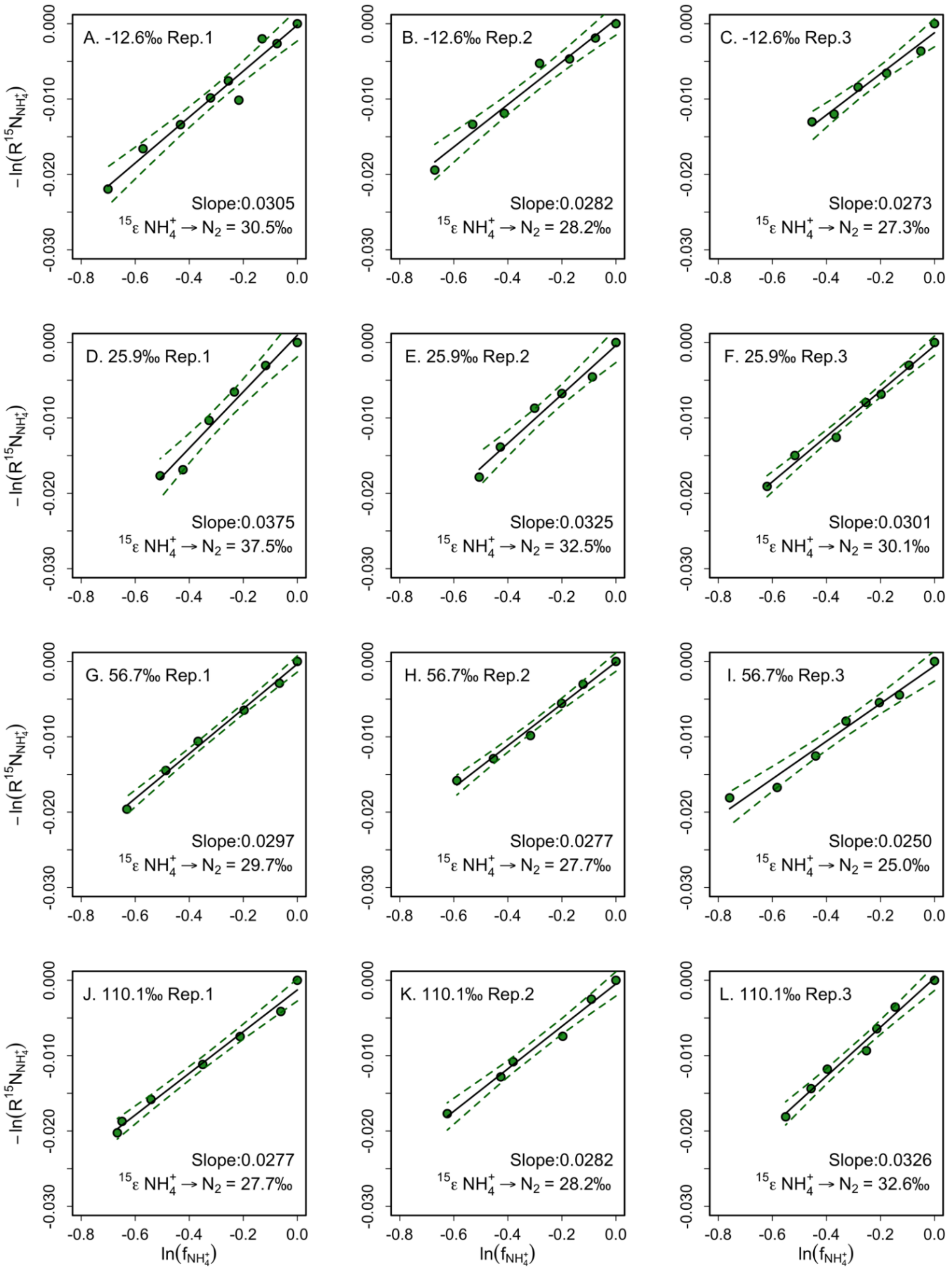

Fig. S5. Estimation of kinetic nitrogen isotope effect ($^{15}\varepsilon_{NH4+\rightarrow N2}$) during NH_4^+ oxidation by the closed-system Rayleigh model as previously described [1]. The dashed line indicates 95% credible interval for determination of $^{15}\varepsilon_{NH4+\rightarrow N2.}$

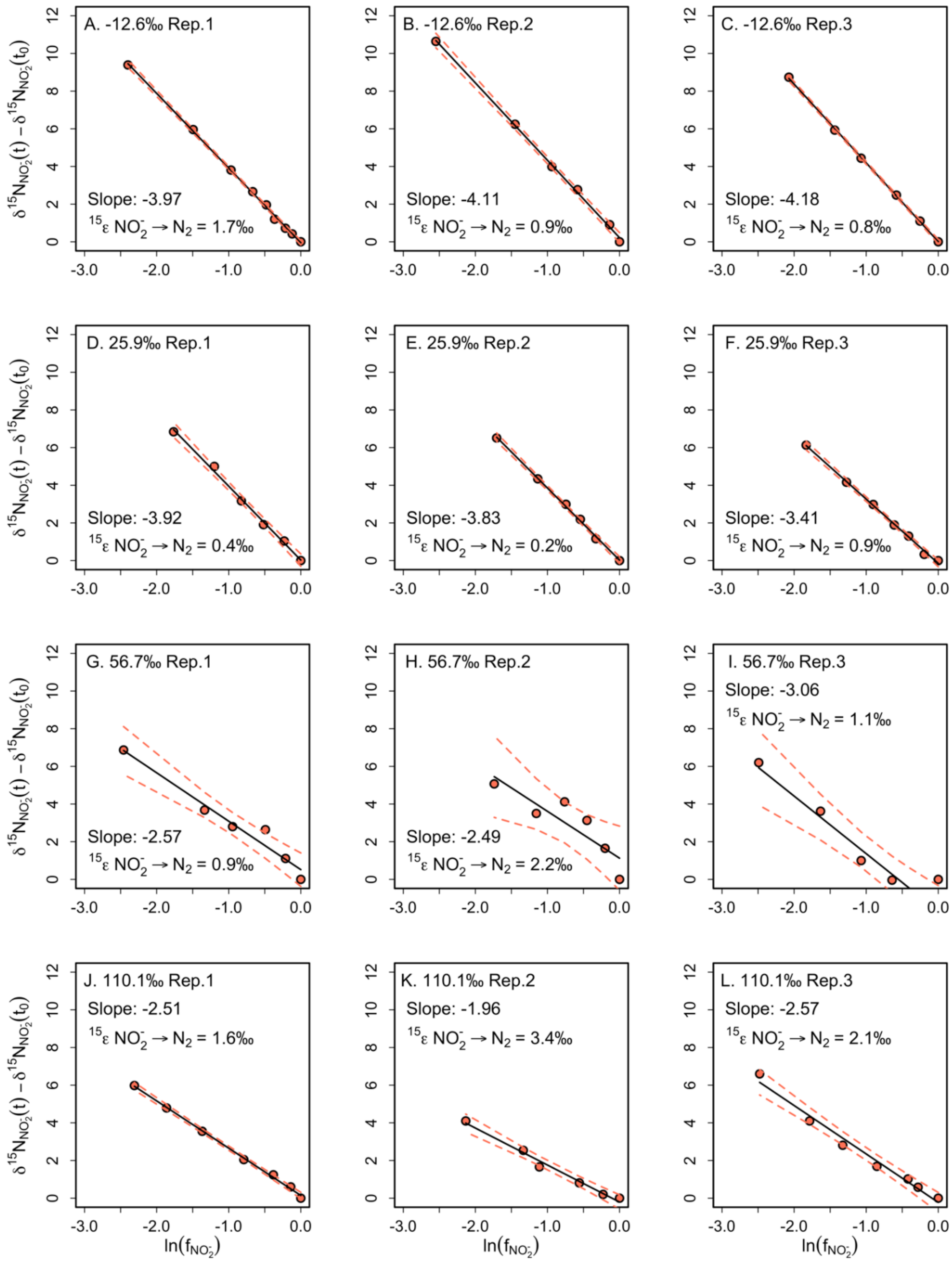

Fig. S6. Estimation of kinetic nitrogen isotope effect ($^{15}\varepsilon_{NO2-\rightarrow N2}$) during NO_2^- reduction by the closed-system Rayleigh model as previously described [1]. The dashed line indicates 95% credible interval for determination of $^{15}\varepsilon_{NO2-\rightarrow N2}$.

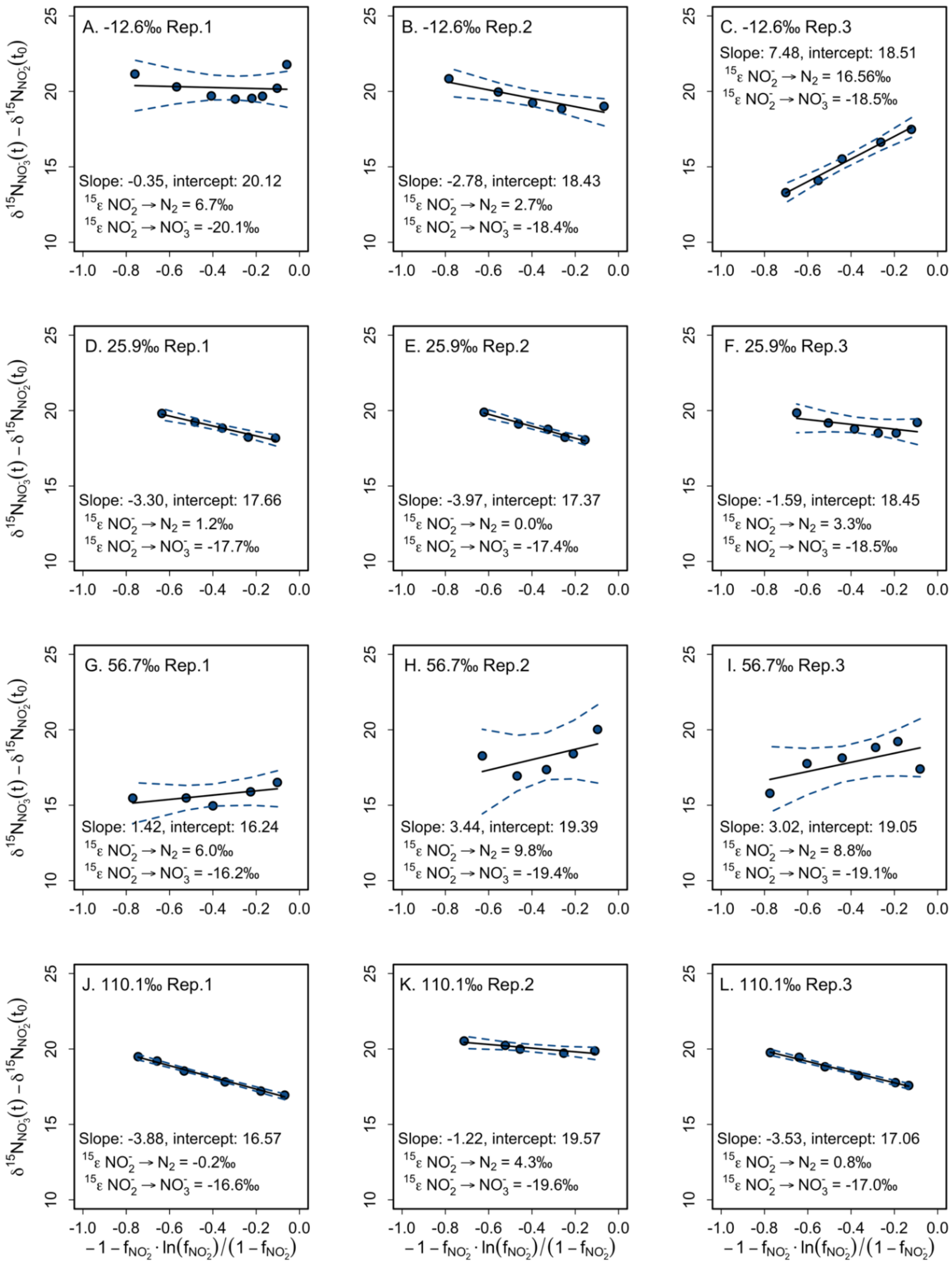

Fig. S7. Estimation of kinetic nitrogen isotope effects ($^{15}\varepsilon_{NO2-\rightarrow N2}$ and $^{15}\varepsilon_{NO2-\rightarrow NO3-}$) during NO_2^- reduction and oxidation by the closed-system Rayleigh model as previously described [1]. The dashed line indicates 95% credible interval for determination of $^{15}\varepsilon_{NO2-\rightarrow N2}$ and $^{15}\varepsilon_{NO2-\rightarrow NO3-}$.

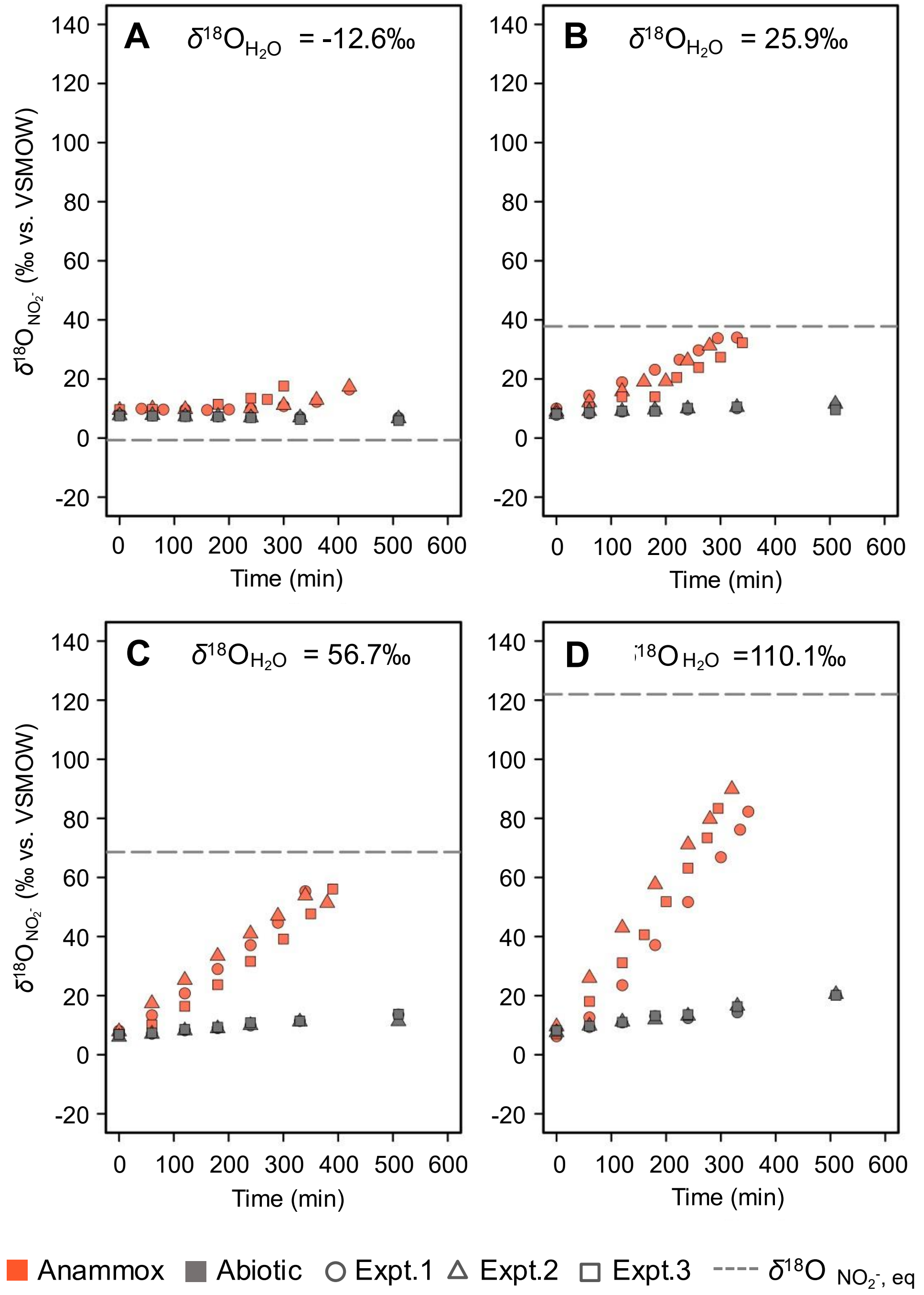

Fig. S8. Comparison of changes in $\delta^{18}O_{NO2-}$ under abiotic conditions (*35*) and during anammox reaction (this study). Triplicate experimental data obtained under $\delta^{18}O_{H2O}$ = -12.6‰ (**A**), $\delta^{18}O_{H2O}$ = 25.9‰ (**B**), $\delta^{18}O_{H2O}$ = 56.7‰ (**C**) and $\delta^{18}O_{H2O}$ = 110.1‰ (**D**) were plotted in respective figures. Dotted lines represent the value of NO_2^- and H_2O equilibrium ($\delta^{18}O_{NO2-, eq}$) of media.

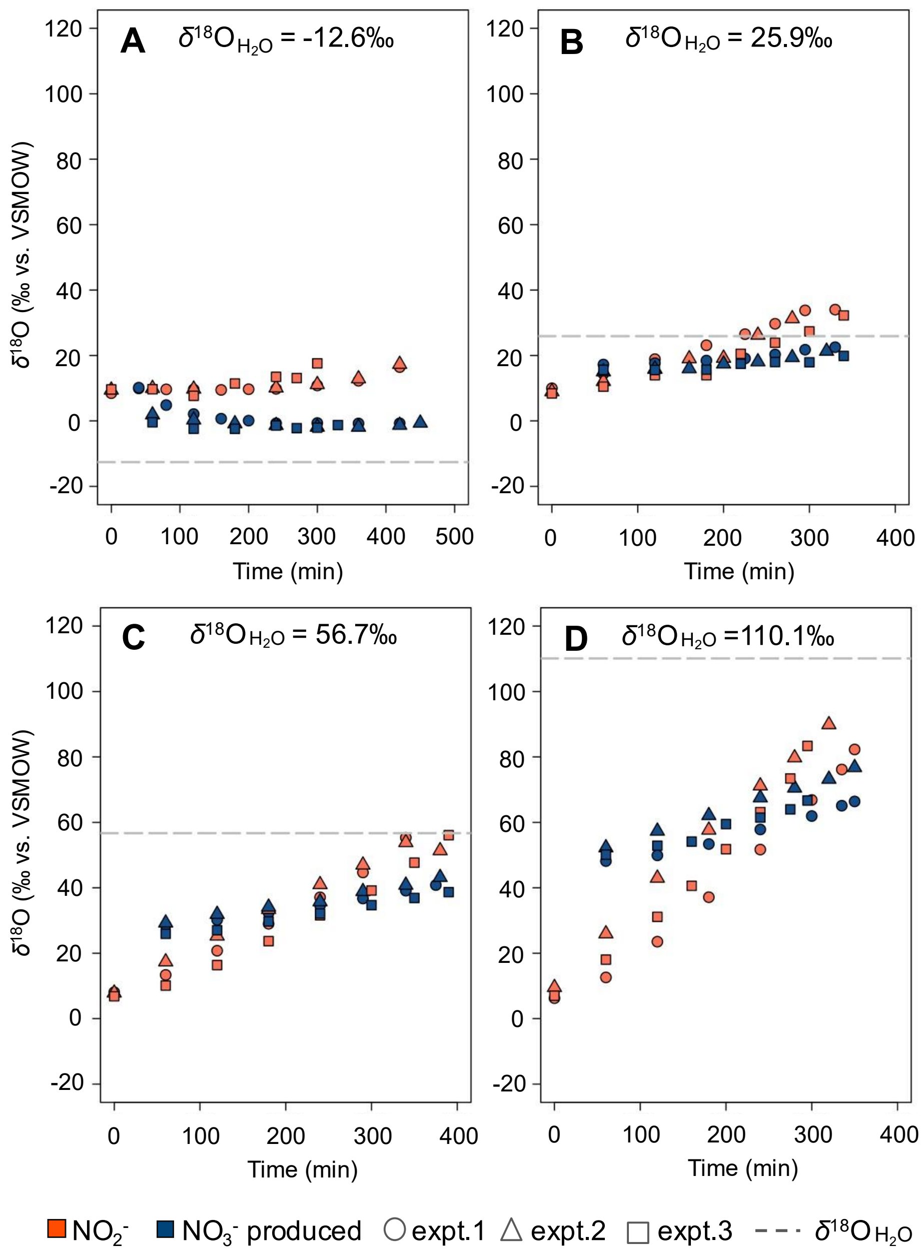

Fig. S9. The dynamic relationships of the δ^{18}O of NO_2^-, produced NO_3^- and H_2O during anammox batch culture experiments with different $\delta^{18}O_{H2O}$ of growth media. Triplicate experimental data obtained under $\delta^{18}O_{H2O}$ = -12.6‰ (**A**), $\delta^{18}O_{H2O}$ = 25.9‰ (**B**), $\delta^{18}O_{H2O}$ = 56.7‰ (**C**) and $\delta^{18}O_{H2O}$ = 110.1‰ (**D**) were plotted in respective figures. We conducted the batch culture experiments in triplicates for each $\delta^{18}O_{H2O}$ media (1st: circle, 2nd: triangle, 3rd: square). Dotted lines represent the value of $\delta^{18}O_{H2O}$ of media. The value of $\delta^{18}O_{NO3\text{- produced}}$ was calculated by following equation：$\delta^{18}O_{NO3\text{- produced}} = (C_{t=t} \times \delta^{18}O_{NO3\text{- }t=t} - C_{t=0} \times \delta^{18}O_{NO3\text{- }t=0}) / (C_{t=t} - C_{t=0})$. According to this equation, the $\delta^{18}O_{NO3\text{-}}$ produced at t = 0 cannot be plotted because it is calculated from the difference from the initial value.

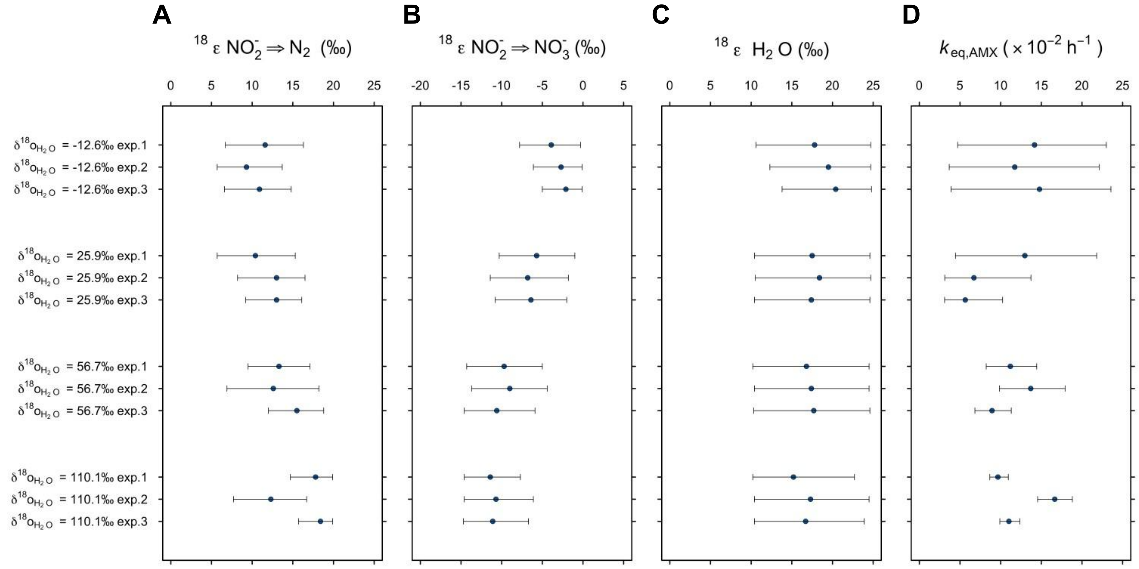

Fig. S10. Estimated oxygen isotope effects ($^{18}\varepsilon$) and their 95% confidence intervals for batch culture experiments with different $\delta^{18}O_{H2O}$ media (**A**:$^{18}\varepsilon_{NO2-\rightarrow N2}$, **B**: $^{18}\varepsilon_{NO2-\rightarrow NO3-}$, **C**: $^{18}\varepsilon_{H2O}$, and **D**: $k_{eq,\ AMX}$). The batch culture experiments were conducted in triplicates for each $\delta^{18}O_{H2O}$ media (exp.1 to 3). The error bars represent 95% confidence interval estimated by the MCMC technique.

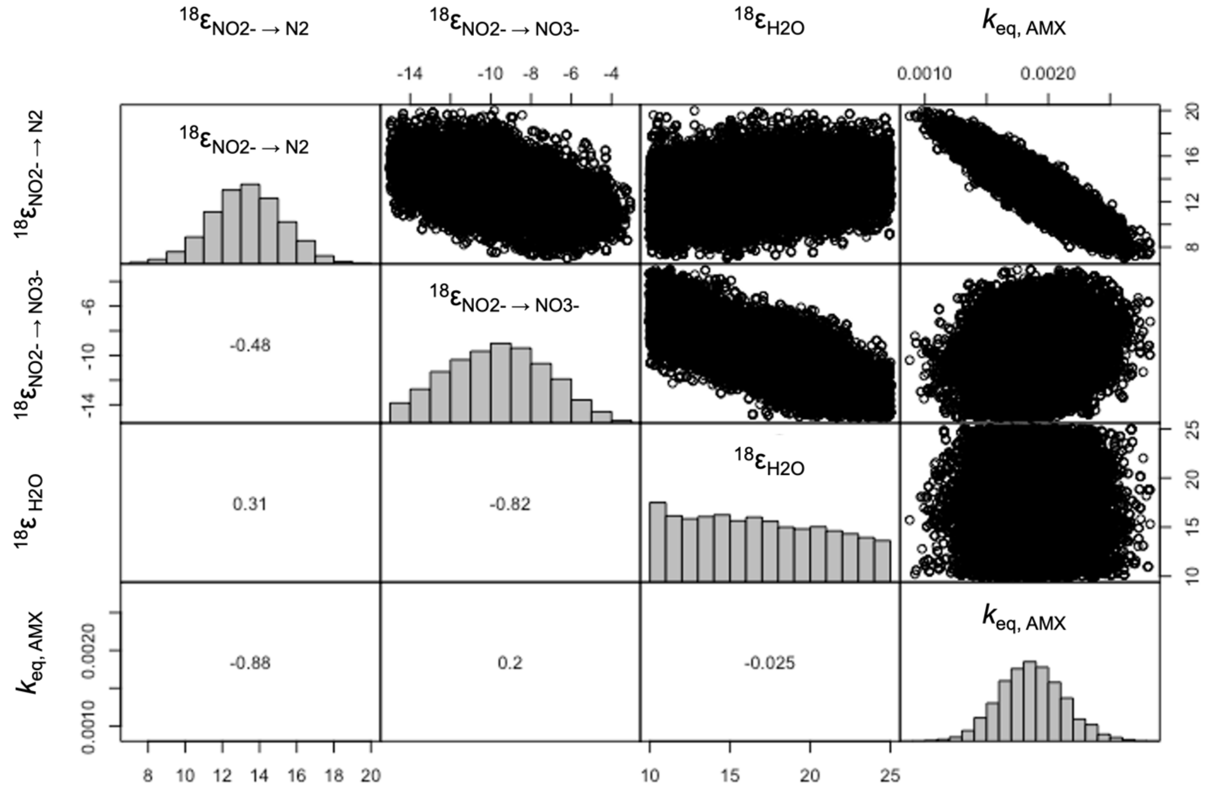

Fig. S11. Pairs plot analyses of each parameter estimated by Markov-Chain Monte-Carlo technique. This figure describes the relationships of each parameter. This representative figure shows the result of first replication experiment under $\delta^{18}O_{H2O}$ =56.7‰

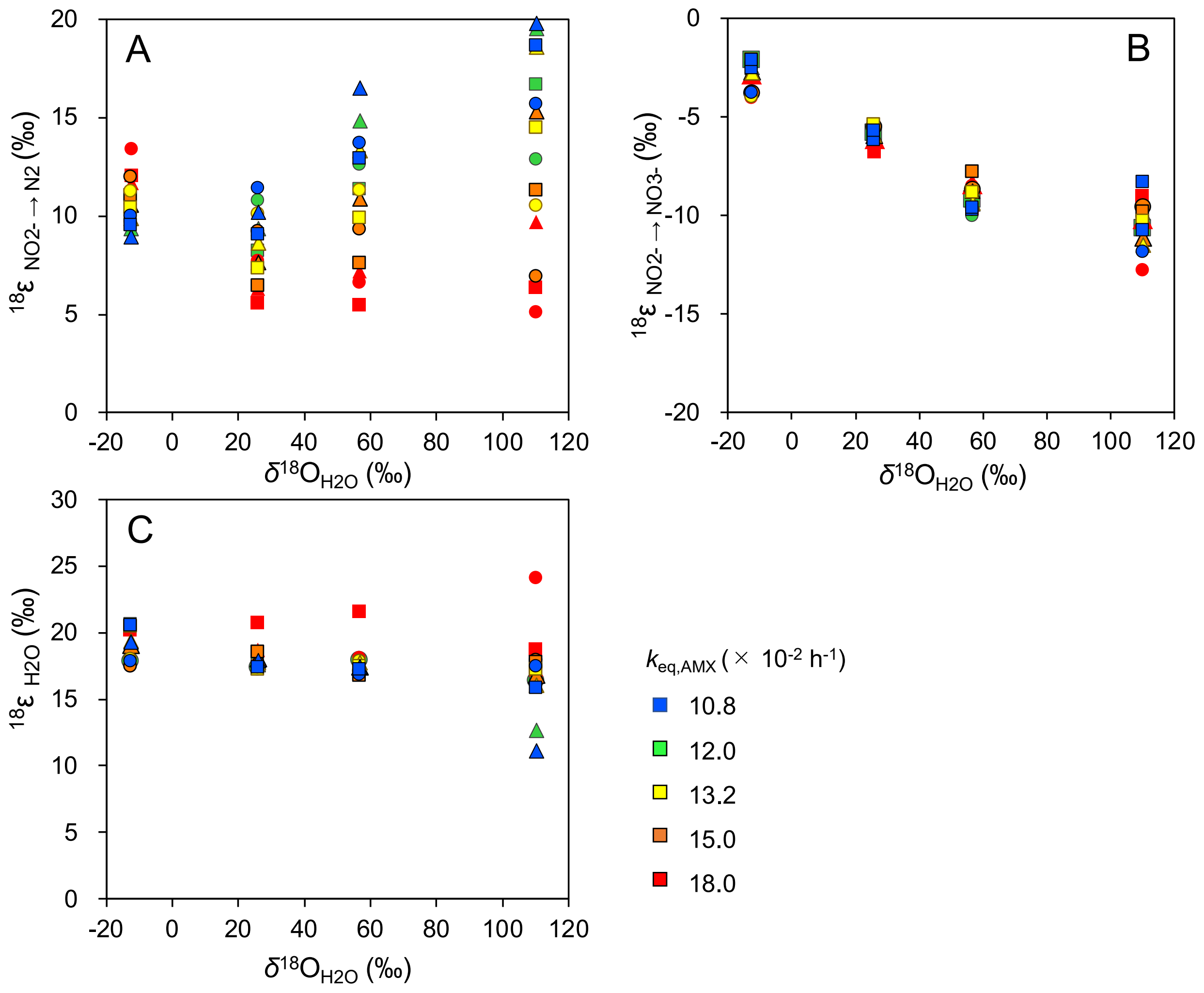

Fig. S12. Effect of $k_{eq,\ AMX}$ on $^{18}\varepsilon_{NO2-\rightarrow N2}$, $^{18}\varepsilon_{NO2-\rightarrow NO3-}$, and $^{18}\varepsilon_{H2O}$. Three parameters (**A**:$^{18}\varepsilon_{NO2-\rightarrow N2}$, **B**: $^{18}\varepsilon_{NO2-\rightarrow NO3-}$, and **C**: $^{18}\varepsilon_{H2O}$) were estimated at different $\delta^{18}O_{H2O}$ media when the value of $k_{eq,\ AMX}$ is fixed at 10.8, 12.0, 13.2, 15.0, and 18.0 ($\times 10^{-2}$ h^{-1}), respectively.

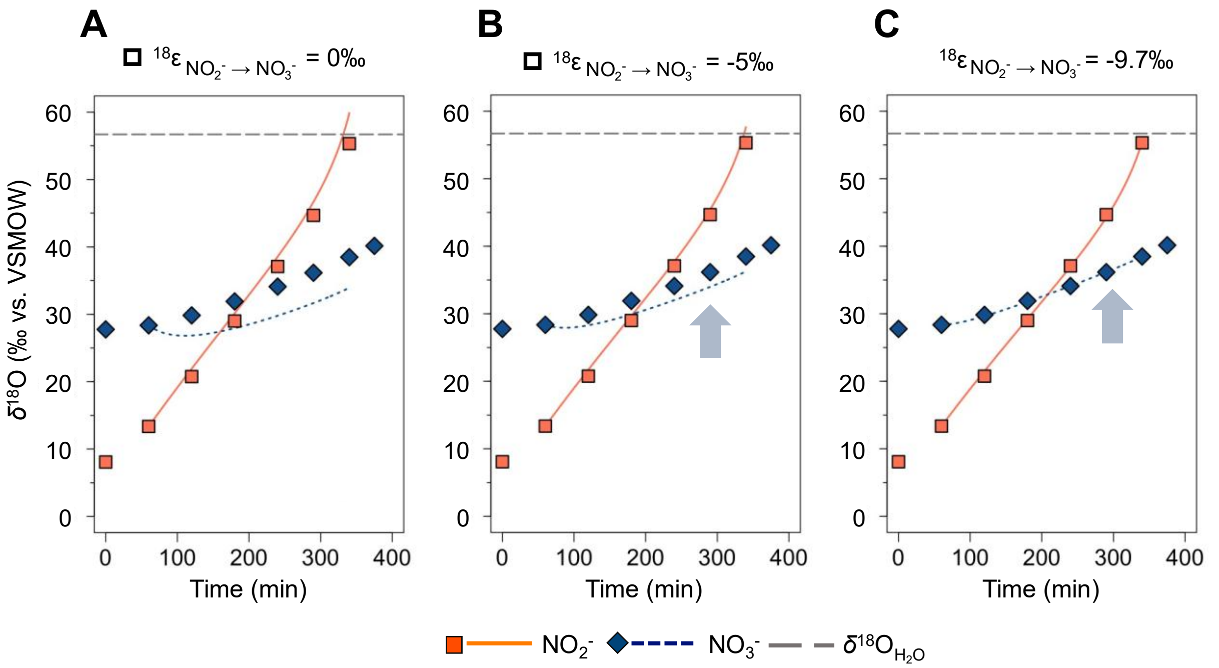

Fig. S13. Effect of the value of $^{18}\varepsilon_{NO2-\rightarrow NO3-}$ on curve fitting of $\delta^{18}O_{NO2-}$ and $\delta^{18}O_{NO3-}$. The representative curve fitting results (under $\delta^{18}O_{H2O}$ = 56.7‰, replication 1) were shown when the value of $^{18}\varepsilon_{NO2-\rightarrow NO3-}$ is fixed at 0‰ (**A**), -5‰ (**B**), or -9.7‰ (**C**), respectively. Other parameters were set as follows; $^{18}\varepsilon_{NO2-\rightarrow N2}$ = 13.3‰, $^{18}\varepsilon_{H2O}$ = 16.8‰, and $k_{eq,\,AMX} = 11.19 \times 10^{-2}\ h^{-1}$(**Table S1B**).

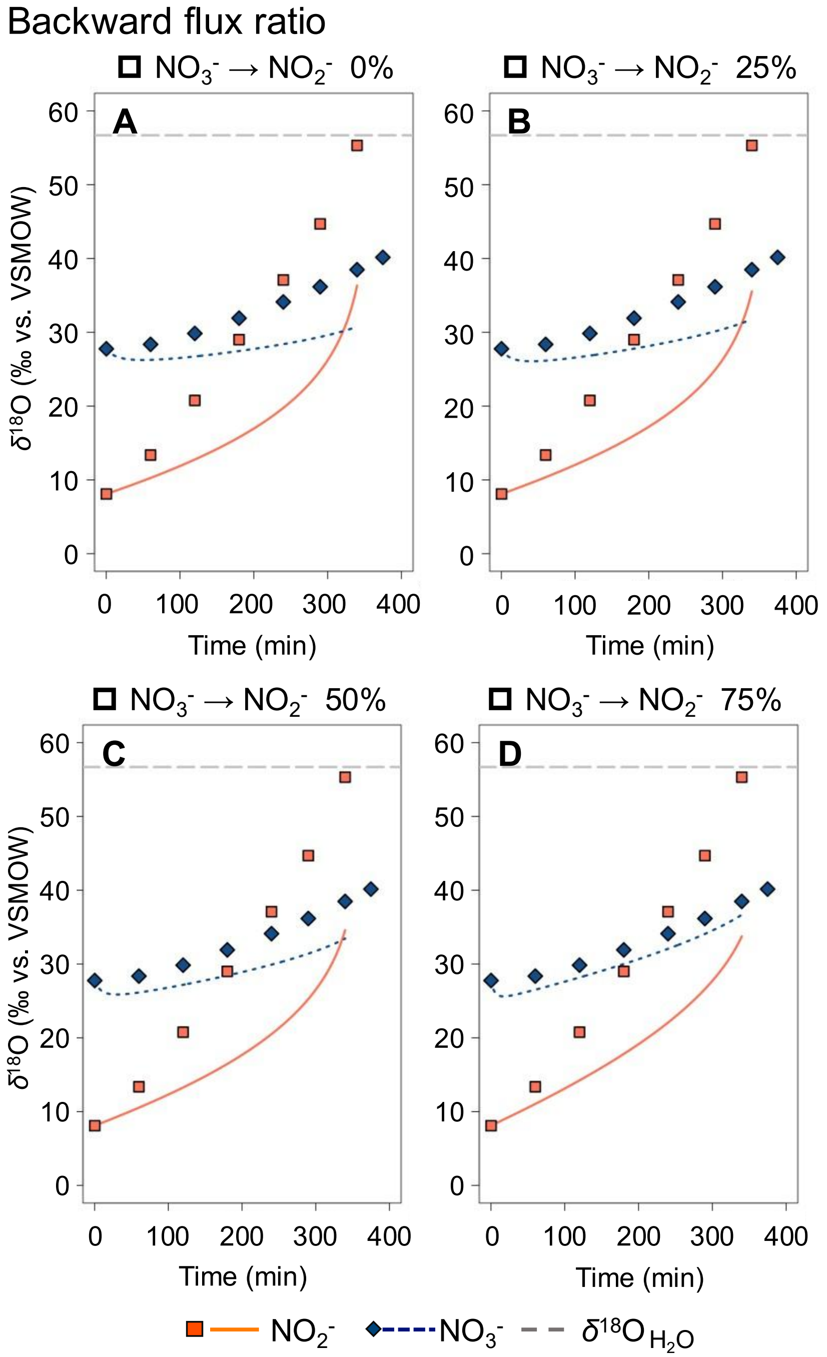

Fig. S14. The effect of reversibility of NO_2^- oxidation reaction (backward flux) on curve fitting of $\delta^{18}O_{NO2-}$ and $\delta^{18}O_{NO3-}$. The representative curve fitting results (under $\delta^{18}O_{H2O}$ = 56.7‰, replication 1) were shown when the ratio of NO_3^- reduction rate (backward flux) to NO_2^- oxidation rate (forward flux) is fixed at 0% (**A**), 25% (**B**), 50% (**C**) and 75% (**D**), respectively. Other parameters were set as follows; $^{18}\varepsilon_{NO2-\rightarrow N2}$ = 13.3‰, $^{18}\varepsilon_{NO2-\rightarrow NO3-}$ = -9.7‰, $^{18}\varepsilon_{H2O}$ = 16.8‰. In this model simulation, parameter of $k_{eq,\ AMX}$ was not included to purely estimate the effect of reversibility of NO_2^- oxidation.

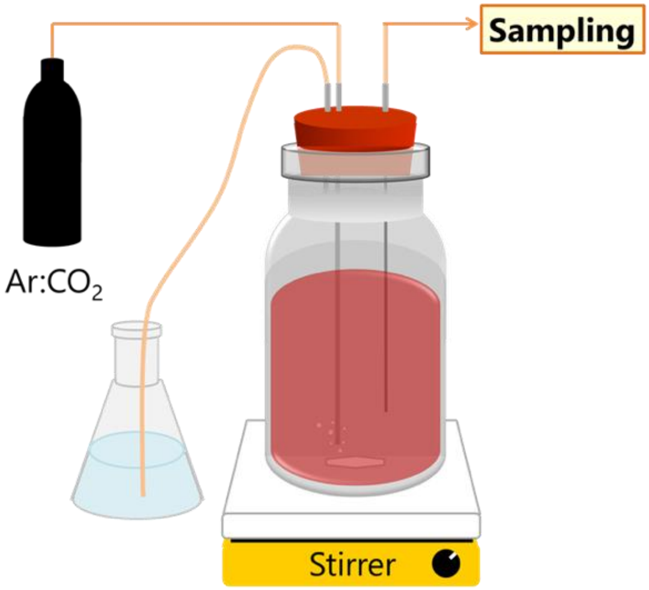

Fig. S15. Schematic drawing of a batch culture experimental set-up.

Table S1. Summary of nitrogen (**A**) and oxygen (**B**) isotope effects determined from batch culture experiments with different $\delta^{18}O_{H2O}$ media by using a newly developed numerical model. The values are the overall mean and SD of the posteriors obtained by each batch experiment.

A

$\delta^{18}O_{H2O}$ of medium	Replication	$^{15}\varepsilon_{NH4+\rightarrow N2}$ (‰)	$^{15}\varepsilon_{NO2-\rightarrow N2}$ (‰)	$^{15}\varepsilon_{NO2-\rightarrow NO3-}$ (‰)
−12.6‰	1	33.8 ± 1.4	11.8 ± 0.6	−17.5 ± 0.6
	2	27.5 ± 1.5	11.7 ± 0.6	−17.2 ± 0.8
	3	29.4 ± 2.1	11.5 ± 0.7	−16.0 ± 0.9
25.9‰	1	36.8 ± 1.9	10.8 ± 0.8	−17.1 ± 1.0
	2	33.5 ± 1.9	10.8 ± 0.9	−17.4 ± 1.0
	3	30.9 ± 1.5	9.2 ± 0.7	−16.5 ± 0.8
56.7‰	1	29.6 ± 1.6	8.1 ± 0.6	−16.7 ± 0.9
	2	29.4 ± 1.8	9.9 ± 0.8	−19.0 ± 1.0
	3	27.8 ± 1.4	7.1 ± 0.5	−17.0 ± 0.8
110.1‰	1	29.0 ± 1.2	8.2 ± 0.5	−17.3 ± 0.8
	2	29.1 ± 1.7	8.1 ± 0.6	−18.4 ± 0.9
	3	34.6 ± 1.8	9.1 ± 0.5	−17.8 ± 0.9

B

$\delta^{18}O_{H2O}$ of medium	Replication	$^{18}\varepsilon_{NO2-\rightarrow N2}$ (‰)	$^{18}\varepsilon_{NO2-\rightarrow NO3-}$ (‰)	$^{18}\varepsilon_{H2O}$ (‰)	$k_{eq, AMX}$ (×10^{-2} h^{-1})
−12.6‰	1	11.6 ± 2.6	− 3.9 ± 2.1	17.8 ± 4.2	14.16 ± 4.93
	2	9.3 ± 2.2	− 2.7 ± 1.7	19.5 ± 3.5	11.73 ± 5.02
	3	10.9 ± 2.2	− 2.1 ± 1.4	20.4 ± 3.1	14.78 ± 5.66
25.9‰	1	10.4 ± 2.6	− 5.7 ± 2.5	17.5 ± 4.3	12.97 ± 4.54
	2	13.0 ± 2.2	− 6.8 ± 2.5	18.4 ± 4.3	6.71 ± 2.89
	3	13.0 ± 1.8	− 6.4 ± 2.4	17.4 ± 4.3	5.65 ± 1.99
56.7‰	1	13.3 ± 1.9	− 9.7 ± 2.5	16.8 ± 4.3	11.19 ± 1.58
	2	12.6 ± 2.9	− 9.0 ± 2.4	17.4 ± 4.2	13.70 ± 2.06
	3	15.5 ± 1.7	− 10.6 ± 2.4	17.7 ± 4.3	8.94 ± 1.14
110.1‰	1	17.8 ± 1.4	− 11.4 ± 1.9	15.2 ± 3.5	9.66 ± 0.59
	2	12.3 ± 2.3	− 10.7 ± 2.3	17.3 ± 4.2	16.65 ± 1.08
	3	18.4 ± 1.2	− 11.1 ± 2.2	16.7 ± 3.9	11.01 ± 0.63

Table S2. Summary of nitrogen isotope effects determined by the closed-system Rayleigh model as previously described [1]. Values represent the mean ± SD of triplicate experimental values of $^{15}\varepsilon$.

δ^{18}O of medium	n	$^{15}\varepsilon_{NH4+\rightarrow N2}$ (‰)	$^{15}\varepsilon_{NO2-\rightarrow N2}$ (‰) by NO_2^- data	$^{15}\varepsilon_{NO2-\rightarrow N2}$ (‰) by NO_3^- data	$^{15}\varepsilon_{NO2-\rightarrow NO3-}$ (‰)
− 12.6‰	3	28.7 ± 1.7	1.1 ± 0.5	8.6 ± 7.2	− 19.0 ± 1.0
25.9‰	3	33.3 ± 3.8	0.5 ± 0.4	1.5 ± 1.7	− 17.8 ± 0.6
56.7‰	3	27.5 ± 2.4	1.4 ± 0.7	8.2 ± 2.0	− 18.2 ± 1.7
110.1‰	3	29.5 ± 2.7	2.4 ± 0.9	1.7 ± 2.4	− 17.7 ± 1.6

Table S3. Description of terms used in modeling equations.

Term	Description
$[NH_4^+]_{initial}$	Initial ammonium concentration (mM)
$[NO_2^-]_{initial}$	Initial nitrite concentration (mM)
$[NO_3^-]_{initial}$	Initial nitrate concentration (mM)
$\delta^{15}N_{NH4,initial}$	Initial nitrogen isotope composition of ammonium (‰ vs. air)
$\delta^{15}N_{NO2,initial}$	Initial nitrogen isotope composition of nitrite (‰ vs. air)
$\delta^{18}O_{NO2,initial}$	Initial oxygen isotope composition of nitrite (‰ vs. VSMOW)
$\delta^{18}O_{NO2,eq}$	The value of $\delta^{18}O_{NO2-}$ at equilibrium state
$\delta^{15}N_{NO3,initial}$	Initial nitrogen isotope composition of nitrate (‰ vs. air)
$\delta^{18}O_{NO3,initial}$	Initial oxygen isotope composition of nitrate (‰ vs. VSMOW)
$\delta^{18}O_{H2O}$	Oxygen isotope composition of ambient water (‰ vs. VSMOW)
$^{14}NAMO$	^{14}N flux of NH_4^+ oxidation to N_2
$^{14}NNIR$	^{14}N flux of NO_2^- reduction to N_2
$^{14}NNXR$	^{14}N flux of NO_2^- oxidation to NO_3^-
$^{15}NAMO$	^{15}N flux of NH_4^+ oxidation to N_2
$^{15}NNIR$	^{15}N flux of NO_2^- reduction to N_2
$^{15}NNXR$	^{15}N flux of NO_2^- oxidation to NO_3^-
$^{16}ONIR$	^{16}O flux of NO_2^- reduction to N_2
$^{16}ONXR$	^{16}O flux of NO_2^- oxidation to NO_3^-
$^{16}ONXR, H_2O$	^{16}O flux of oxygen incorporation from H_2O during NO_2^- oxidation to NO_3^-
$^{18}ONIR$	^{18}O flux of NO_2^- reduction to N_2
$^{18}ONXR$	^{18}O flux of NO_2^- oxidation to NO_3^-
$^{18}ONXR, H_2O$	^{18}O flux of oxygen incorporation from H_2O during NO_2^- oxidation to NO_3^-
$^{18}O_{NO2-}(DT)_{exchange}$	Change of ^{18}O pool due to abiotic oxygen isotope exchange between NO_2^- and H_2O
$^{18}O_{NO2-}(DT)_{exchange, AMX}$	Change of ^{18}O pool due to anammox-mediated oxygen isotope exchange between NO_2^- and H_2O

Table S4. Description of parameters and their estimation ranges used in model simulation.

Parameters	Description	Range
$^{15}\varepsilon_{AMO}$	Nitrogen isotope effect of NH_4^+ oxidation to N_2	−60 to 60 (‰)
$^{15}\varepsilon_{NIR}$	Nitrogen isotope effect of NO_2^- reduction to N_2	−60 to 60 (‰)
$^{15}\varepsilon_{NXR}$	Nitrogen isotope effect of NO_2^- oxidation to NO_3^-	−60 to 60 (‰)
$^{18}\varepsilon_{NIR}$	Oxygen isotope effect of NO_2^- reduction to N_2	5 to 20 (‰)
$^{18}\varepsilon_{NXR}$	Oxygen isotope effect of NO_2^- oxidation to NO_3^-	−15 to 0 (‰)
$^{18}\varepsilon_{NXR,\ H2O}$	Oxygen isotope effect of oxygen incorporation from H_2O during NO_2^- oxidation to NO_3^-	10 to 25 (‰)
$k_{\mathrm{eq,\ AMX}}$	reaction rate constant of anammox-mediated oxygen isotope exchange between NO_2^- and H_2O	3.0 to 24 ($\times 10^{-2}$ h^{-1})

Table S5. The parameter values used in anammox model exercises to evaluate the impact of $\delta^{18}O_{H2O}$ on the $\Delta\delta^{18}O$: $\Delta\delta^{15}N$ trajectory of NO_2^- and NO_3^- during anammox reaction.

Term	Description	Value	Reference
$[NH_4^+]_{initial}$	Initial ammonium concentration (mM)	2.62	This study
$[NO_2^-]_{initial}$	Initial nitrite concentration (mM)	1.87	This study
$[NO_3^-]_{initial}$	Initial nitrate concentration (mM)	0.03	This study
$\delta^{15}N_{NH4,initial}$	Initial nitrogen isotope composition of ammonium (‰ vs. air)	5	Granger and Wankel 2016
$\delta^{15}N_{NO2,initial}$	Initial nitrogen isotope composition of nitrite (‰ vs. air)	0	5‰ lower than $\delta^{15}N_{NO3-}$
$\delta^{18}O_{NO2,initial}$	Initial oxygen isotope composition of nitrite (‰ vs. VSMOW)	9	Around $\delta^{18}O_{NO2-,eq}$
$\delta^{15}N_{NO3,initial}$	Initial nitrogen isotope composition of nitrate (‰ vs. air)	5	Sigman & Fripat 2019
$\delta^{18}O_{NO3,initial}$	Initial oxygen isotope composition of nitrate (‰ vs. VSMOW)	2	Sigman & Fripat 2019
$\delta^{18}O_{H2O}$	Oxygen isotope composition of ambient water (‰ vs. VSMOW)	−7.7 ~ 1.8	Bowen et al., 2010
$^{15}\varepsilon_{NH4+\rightarrow N2}$	N isotope effect for NH_4^+ oxidation (‰)	30.7	This study
$^{15}\varepsilon_{NO2-\rightarrow N2}$	N isotope effect for NO_2^- reduction (‰)	9.5	This study
$^{15}\varepsilon_{NO2-\rightarrow NO3-}$	N isotope effect for NO_2^- oxidation (‰)	-17.4	This study
$^{18}\varepsilon_{NO2-\rightarrow N2}$	O isotope effect for NO_2^- reduction (‰)	10.6	This study (result of $\delta^{18}O_{H2O}$ = -12.6‰)
$^{18}\varepsilon_{NO2-\rightarrow NO3-}$	O isotope effect for NO_2^- oxidation (‰)	-2.9	This study (result of $\delta^{18}O_{H2O}$ = -12.6‰)
$^{18}\varepsilon_{H2O}$	O isotope effect for O atom incorporation during NO_2^- oxidation(‰)	19.2	This study (result of $\delta^{18}O_{H2O}$ = -12.6‰)
$k_{eq,\ AMX}$	Reaction rate constant of anammox-mediated oxygen isotope exchange between NO_2^- and H_2O ($\times 10^{-2}$ h^{-1})	13.56	This study (result of $\delta^{18}O_{H2O}$ = -12.6‰)

Reference

1. Brunner B et al. Nitrogen isotope effects induced by anammox bacteria. *Proc Natl Acad Sci U. S. A* 2013;**110**:18994–9. 10.1073/pnas.1310488110
